# Direct solvothermal growth of morphology-controlled TaS_2_ on substrates enables stable self-supported hydrogen evolution electrocatalysts

**DOI:** 10.1039/d6ta07594k

**Published:** 2026-10-26

**Authors:** Cecilia A. Zito, Francesco Caddeo, Philipp M. Ulmen, Emil Schürmann, Yvonne Pieper, Jagadesh Kopula Kesavan, Heshmat Noei, Francesca M. Toma, Andreas Stierle, Dorota Koziej

**Affiliations:** a University of Hamburg, Institute for Nanostructure and Solid-State Physics, Center for Hybrid Nanostructures Luruper Chaussee 149 22761 Hamburg Germany cecilia.zito@uni-hamburg.de dorota.koziej@uni-hamburg.de; b The Hamburg Centre for Ultrafast Imaging Hamburg Germany; c Institute of Functional Materials for Sustainability, Helmholtz-Zentrum Hereon Kantstrasse 55 14513 Teltow Germany; d Faculty of Mechanical and Civil Engineering, Helmut Schmidt University Holstenhofweg 85 22043 Hamburg Germany; e Centre for X-Ray and Nano Science CXNS, Deutsches Elektronen-Synchrotron DESY Notkestrasse 85 22607 Hamburg Germany; f Institute for Nanostructure and Solid-State Physics, University of Hamburg Hamburg Germany

## Abstract

Layered transition metal dichalcogenides represent a class of materials with promising electronic properties and emerging applications as electrocatalysts for the hydrogen evolution reaction (HER). However, their preparation relies on vapor-phase and solid-state methods, while solution-phase synthesis remains challenging and underdeveloped. Here, a solvothermal route is demonstrated for the direct growth of TaS_2_ on different substrates. The reaction between TaCl_5_ and 1-dodecanethiol in toluene yields highly crystalline TaS_2_ nanostructures, whose morphology can be tailored from nanodisks (NDs) to nanosheets (NSs) through the addition of octylamine while preserving a single-phase 1T structure. Direct growth on conductive substrates, including Ta plate and carbon cloth, enables the fabrication of self-supported electrodes, which, when tested for HER under acidic conditions, reveal that both catalyst morphology and the underlying substrate influence electrocatalytic performance. Among the electrodes, TaS_2_ NSs grown on the Ta plate exhibit the highest activity, demonstrating that the developed synthetic approach enables morphological control to tune catalytic activity. Moreover, all self-supported electrodes show excellent long-term stability regardless of morphology or substrate. Overall, this work establishes a versatile solvothermal strategy for growing morphology-controlled TaS_2_ nanostructures, enabling their integration into self-supported electrodes while providing a pathway for future investigations of TaS_2_ in energy-related and electronic applications.

## Abstract 2

Solvothermal synthesis enables morphological control of TaS_2_ from nanodisks to nanosheets and their direct growth on substrates, yielding self-supported electrodes with electrocatalytic activity for HER and prolonged stability.

## Graphical abstract

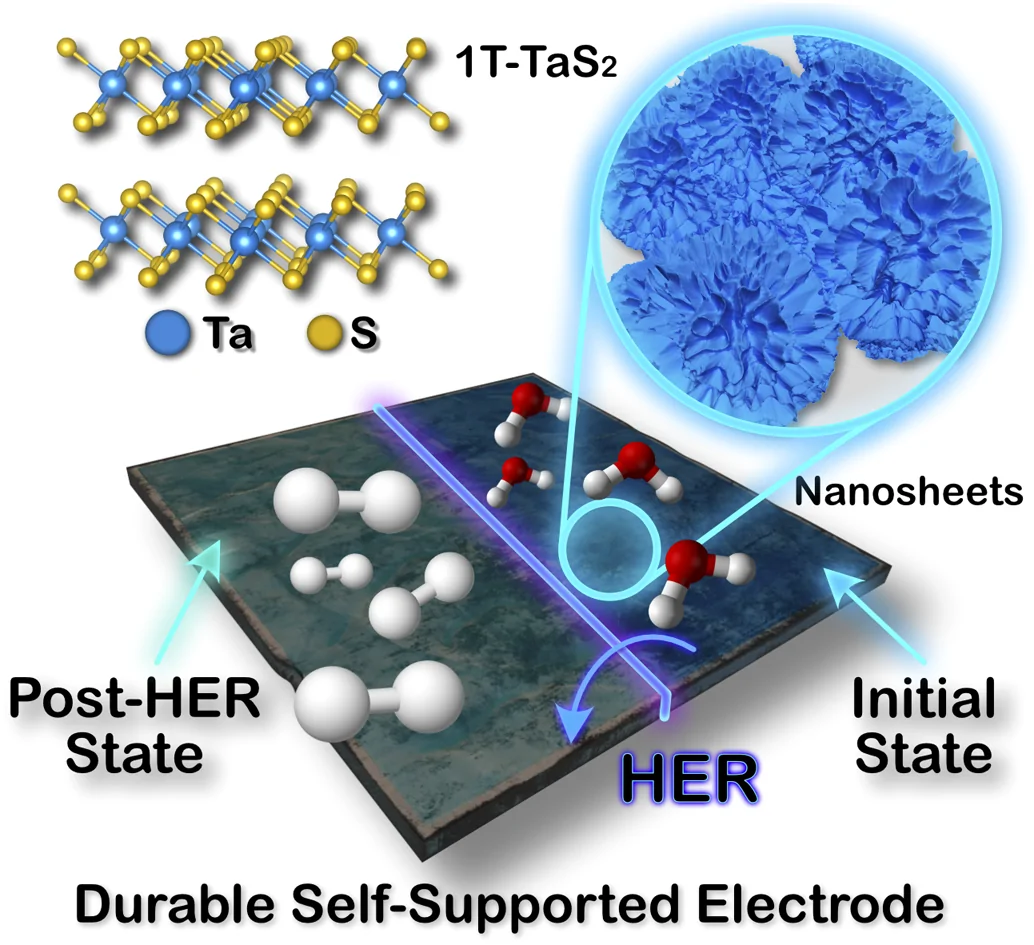

## 1. Introduction

Two-dimensional transition metal dichalcogenides (TMDs) have attracted significant attention due to their unique intrinsic properties and the wide range of applications enabled by their layered structures. Among group V TMDs, TaS_2_ exhibits remarkable phenomena such as charge density wave (CDW) phases,^1,2^ superconductivity,^3,4^ and Mott insulating behavior.^5,6^ Beyond these distinct properties, TaS_2_ and related TMDs have recently emerged as promising alternatives to platinum (Pt) and other noble metals for green hydrogen production *via* the electrocatalytic hydrogen evolution reaction (HER).^7–10^ This is primarily attributed to the large number of active sites provided by the layered structure, the efficient charge-transfer characteristics, and the possibility of enhancing activity through defect and structural modifications.^11,12^

In this context, the electrocatalytic activity of TaS_2_ for the HER has been modulated through various strategies, including plasma etching,^13^ microwave treatment,^14^ interfacial engineering with the substrates,^15^ and phase engineering.^16–18^ Unlike MoS_2_, where the active sites are predominantly localized at edge sites, both basal planes and edge sites in TaS_2_ have been identified as catalytically active.^19,20^ However, the practical implementation of TaS_2_ is limited by the lack of simple, scalable, and low-temperature synthesis routes.

To date, the synthesis of TaS_2_ has relied mostly on methods such as chemical vapor deposition (CVD),^15,21^ solid-state reactions in furnaces at high temperature (>800 °C),^17,22^ intercalation and exfoliation of bulk TaS_2_,^23^ chemical vapor transport (CVT),^1,24^ and reactive molecular beam epitaxy (MBE).^25^ Meanwhile, solution-phase synthesis, one of the most powerful routes for size and morphological control in nanomaterials, remains highly challenging for TaS_2_, with very few suitable precursor combinations available. Reported approaches include hot-injection methods at 300 °C using neurotoxic CS_2_ as the sulfur source,^18,26,27^ a heat-up approach in dodecylamine and elemental sulfur at 250 °C that requires post-synthetic heat treatment,^28^ and long solvothermal reactions, which take several days and need subsequent additional heating above 200 °C to achieve full crystallinity of the products.^29^

In contrast to previously reported methods, we employ a straightforward one-step solvothermal approach at relatively mild temperatures (≤230 °C), based on the reaction of TaCl_5_ with 1-dodecanethiol in toluene. The developed method enables morphological control through the use of a small amount of octylamine as an organic ligand, while simultaneously promoting the direct growth of TaS_2_ particles on conductive substrates, including tantalum (Ta) plate and carbon cloth (CC). By employing these substrates, the approach enables the design of self-supported electrodes, which have so far been achieved for TaS_2_ primarily *via* high-temperature vapor-phase methods,^15,30,31^ but not through solution-phase routes. The direct growth of catalytic materials on conductive substrates enhances adhesion, simplifies electrode production, eliminates the need for conductive binders such as Nafion, improves electrode stability, and promotes efficient interfacial charge transfer between the substrate and catalyst.^30,32–35^ Herein, we demonstrate that the direct growth of TaS_2_ nanostructures on conductive substrates yields self-supported electrodes with excellent long-term stability for over 45 h. In particular, the controlled growth of thin TaS_2_ nanosheets on Ta plates results in enhanced HER performance and improved reaction kinetics.

## 2. Results and discussion

### 2.1 Synthesis and structural characterization of TaS_2_

We developed a solvothermal method for the direct growth of 1T-TaS_2_ nanostructures on various conductive substrates, including carbon cloth (CC), a metallic Ta plate, and glassy carbon (GC). This approach enables the fabrication of self-supported TaS_2_-based electrocatalysts, as schematically illustrated in Fig. 1a. Using toluene as a solvent, the reaction between TaCl_5_ and 1-dodecanethiol (1-DDT) in a 1 : 20 molar ratio at 210 °C yields TaS_2_ nanodisks (NDs). When octylamine is used as a ligand in the reaction, combined with an increased temperature of 230 °C, the morphology changes, yielding thin TaS_2_ nanosheets (NSs). To distinguish the effect of the octylamine from that of temperature, a control reaction was carried out on a CC at 230 °C in the absence of octylamine. Despite the increased temperature, NDs remain the predominant morphology (Fig. S1), indicating that the morphological control is associated with the use of octylamine. Notably, we found that both types of TaS_2_ nanostructures can be obtained at reaction times as short as 3 h.

**Fig. 1:**
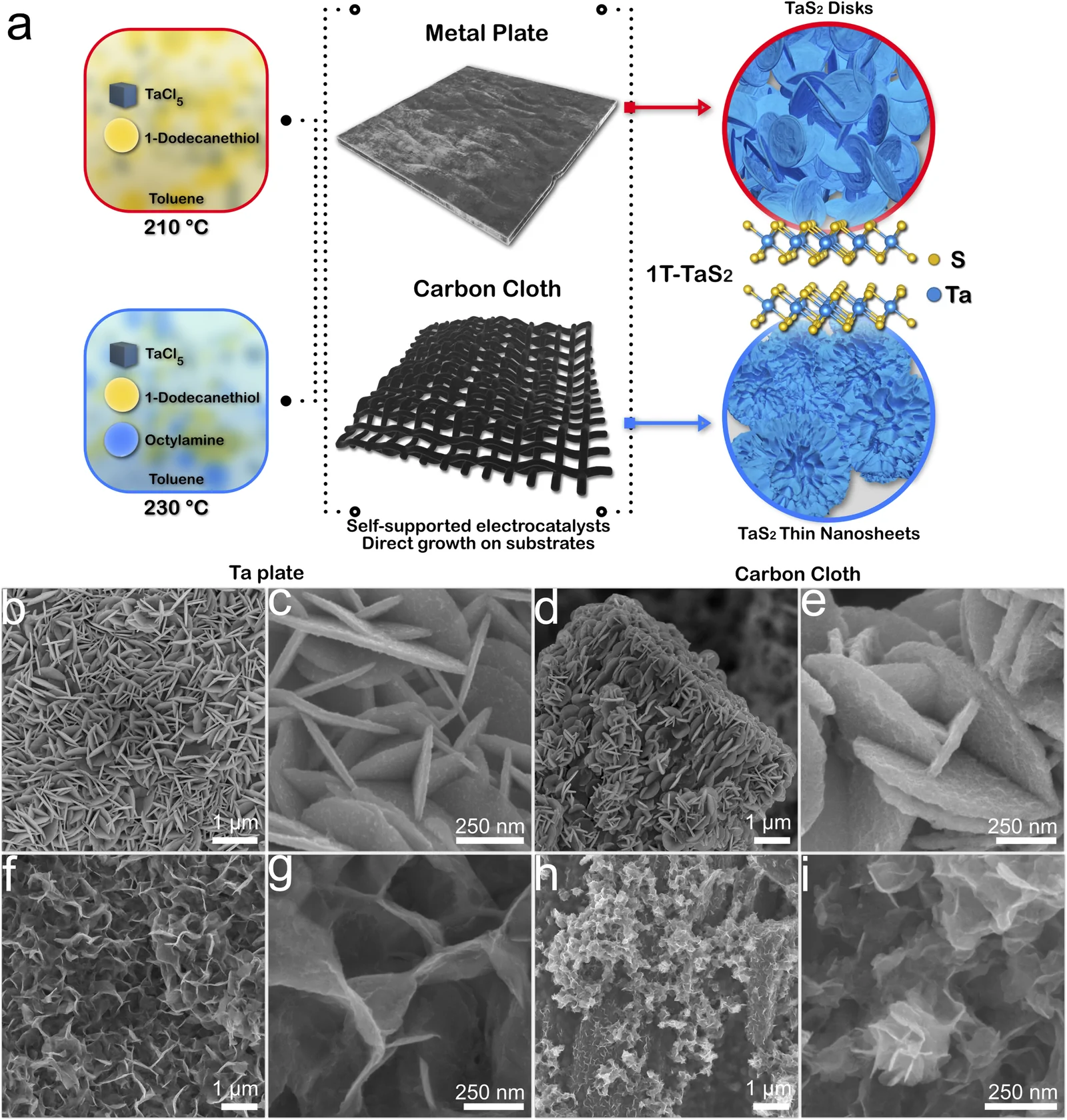
Solvothermal growth of 1T-TaS_2_ nanostructures on conductive substrates. (a) Schematic illustration of the synthesis of TaS_2_ nanodisks (NDs) and thin nanosheets (NSs) on different substrates. (b–i) SEM images of TaS_2_ nanostructures on Ta plate and carbon cloth (CC): (b,c) TaS_2_ NDs on Ta plate synthesized at 210 °C for 3 h; (d,e) TaS_2_ NDs on CC synthesized at 210 °C for 12 h; (f,g) TaS_2_ NSs on Ta plate synthesized at 230 °C for 12 h using octylamine; and (h,i) TaS_2_ NSs on CC obtained at 230 °C for 12 h using octylamine.

Fig. 1b–i exhibit the scanning electron microscopy (SEM) characterization of TaS_2_ NDs and NSs grown on two different substrates, a Ta plate and CC. When the reaction is carried out at 210 °C in the absence of octylamine, we observe the formation of highly interconnected TaS_2_ disk-like nanostructures, along with partially intergrown disk-like features. As evidenced in Fig. 1b and c, using the Ta plate as a substrate results in the formation of a continuous and homogeneous film that uniformly covers the flat surface of the substrate. In contrast, using CC as the substrate leads to the NDs coating the surface of the carbon fibers, following the fiber network, as displayed in Fig. 1d and e. In both cases, the TaS_2_ NDs exhibit a rough, textured surface and comparable dimensions despite the different substrates employed. The NDs have average diameters of ∼550–580 nm and a thickness of ∼27–35 nm. However, due to the highly interconnected and partially vertically oriented growth of these structures, the diameter values should be considered approximate. As shown in Fig. S2, increasing the reaction time from 3 h to 36 h using CC as the substrate preserves the interconnected disk-like morphology throughout the reaction series. Moreover, the loading of TaS_2_ NDs on the CC varies from ∼1.91 mg cm^−2^ after 3 h to 17.3 mg cm^−2^ after 36 h of reaction time. Fig. S3 shows the morphology of the bare CC, which consists of carbon fibers with carbon-particle-filled regions.

In Fig. 1f–i, we highlight the effect of using octylamine in the reaction carried out at 230 °C. The SEM images in Fig. 1f and g elucidate the formation of wrinkled, sheet-like nanostructures of TaS_2_ on the surface of the Ta plate. The TaS_2_ NSs form a continuous, highly interconnected network across the Ta plate surface. As exhibited in Fig. S4, these characteristics are also seen at different reaction times using the Ta plate as the substrate. Similarly, when using CC as the substrate, crumpled TaS_2_ NSs form on the surface of the carbon fibers, resembling flower-like nanostructures, as shown in Fig. 1h and i. The carbon fibers are covered with TaS_2_ NSs at all reaction times, from 3 to 36 h (see Fig. S5). The loading of TaS_2_ NSs on CC is around 0.32 mg cm^−2^ for all reaction times, indicating a less efficient interaction of TaS_2_ NSs with the CC substrate compared to NDs. Besides the direct growth of TaS_2_ NSs on substrates, the reaction performed using octylamine also produces recoverable TaS_2_ powder (Fig. S6). Similar findings are observed when using GC as a substrate, as depicted in Fig. S7.

We employ transmission electron microscopy (TEM) to further study the morphological and atomic structural differences between TaS_2_ NDs and NSs (Fig. 2). Fig. 2a exhibits a low-magnification TEM image of representative TaS_2_ NDs, synthesized using a Ta plate as the substrate. The corresponding selected-area electron diffraction (SAED) pattern in the inset confirms the 1T-TaS_2_ phase of the NDs. The TEM images in Fig. 2a and b reveal the interconnected nature of the TaS_2_ NDs, which assemble into a flower-like structure. The 1T-TaS_2_ phase is further evidenced by the (011) lattice fringes shown in the high-resolution TEM (HRTEM) image (Fig. 2c).

**Fig. 2:**
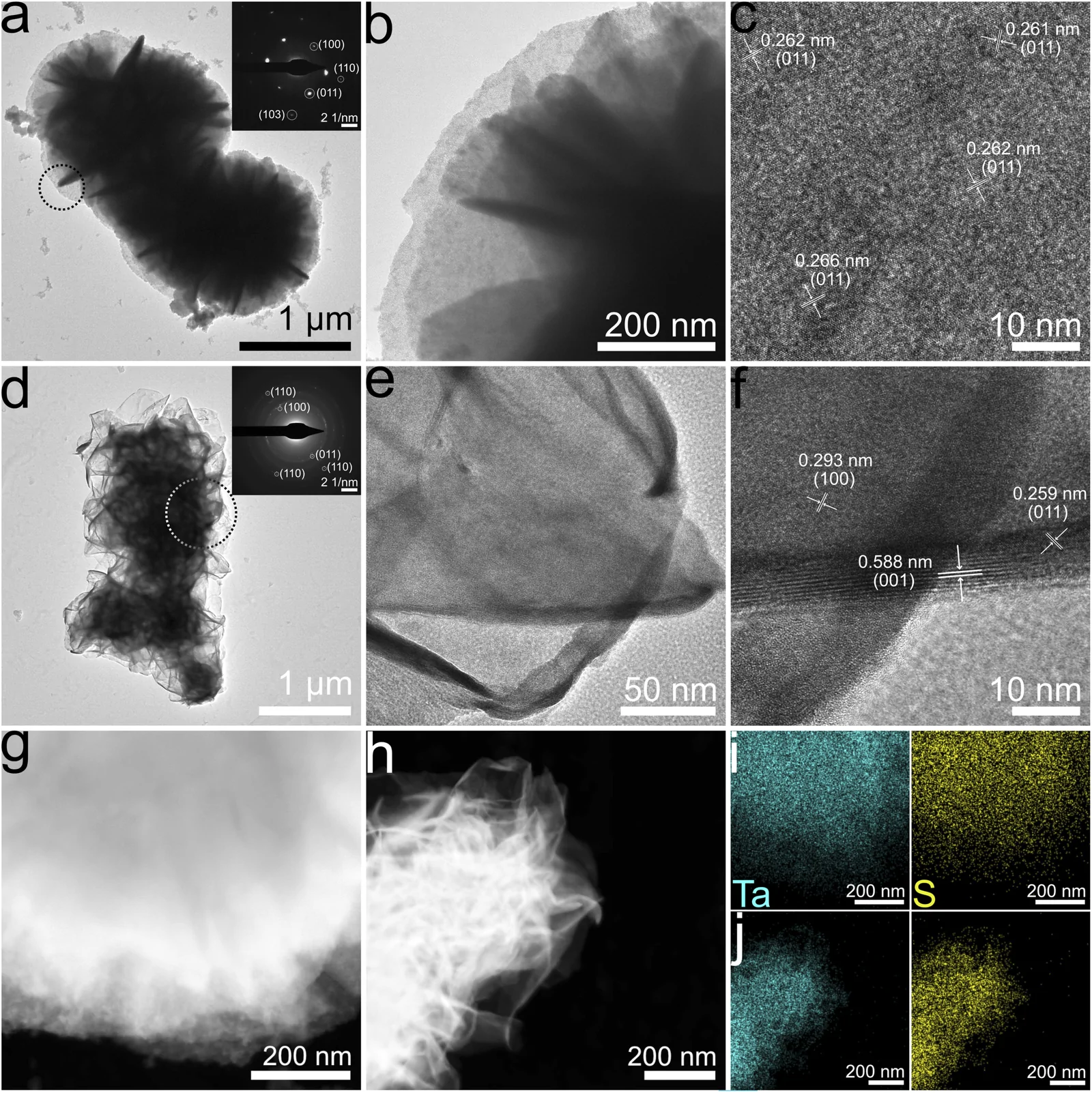
Morphology and structural characterization of TaS_2_ nanostructures grown on a Ta plate. (a–c) TaS_2_ NDs synthesized at 210 °C for 3 h: (a,b) TEM images of the interconnected NDs (inset in (a) shows the SAED pattern of the circled area), and (c) HRTEM image. (d–f) TaS_2_ NSs obtained at 230 °C for 12 h using octylamine: (d,e) TEM images of the thin, crumpled NSs structure (inset in (d) corresponds to the SAED pattern of the circled area), and (f) HRTEM image. (g) HAADF-STEM image of TaS_2_ NDs and (i) corresponding EDX elemental mapping of Ta and S. (h) HAADF-STEM image of TaS_2_ NSs and (j) corresponding EDX elemental mapping of Ta and S.

As shown in Fig. 2d, the TEM image of a representative assembly of crumpled TaS_2_ NSs grown on a Ta plate reveals the ultrathin nature of the NSs, reflected by their high transparency to the beam. The crumpled morphology results in numerous exposed sheet edges. The 1T-TaS_2_ phase is evidenced in the corresponding SAED pattern (inset of Fig. 2d). The higher-magnification TEM image highlights the folding behavior of the NSs, see Fig. 2e. Such behavior allows us to observe the layered structure of the material, with an interlayer spacing of ∼0.588 nm, corresponding to the (001) plane of the 1T phase, as shown in the HRTEM image in Fig. 2f. Additional lattice fringes with *d*-spacings of 0.293 nm and 0.259 nm, corresponding to the (100) and (011) planes of TaS_2_, respectively, are also observed. Fig. 2g and h show the high-angle annular dark-field scanning TEM (HAADF-STEM) images of TaS_2_ NDs and NSs, respectively. The corresponding energy-dispersive X-ray spectroscopy (EDX) mapping confirms the even distribution of Ta and S across both nanostructures, as depicted in Fig. 2i and j. Additional TEM characterization for TaS_2_ NDs and NSs grown on CC is given in Fig. S8.

We employ powder X-ray diffraction (PXRD) to evaluate the crystallinity and phase composition of both nanostructures grown on the CC and Ta plate. In Fig. 3a, we observe that the sample based on the NDs obtained after 12 h of solvothermal growth on CC exhibits intense diffraction peaks corresponding to the 1T phase of TaS_2_ (space group *P*3̄*m*1, No. 164; ICSD: 85323),^36^ with only additional reflections originating from the substrate. This indicates a high degree of crystallinity and phase purity. In contrast, the PXRD pattern of the TaS_2_ grown on the Ta plate is mostly dominated by reflections from the metallic Ta substrate. Yet, a magnified view of the diffraction region between 5° and 37° clearly reveals the diffraction peaks corresponding to 1T-TaS_2_, despite the thin nature of the NSs (inset of Fig. 3a). PXRD patterns of the NDs and NSs grown on the CC and Ta plate using different reaction times are provided in Fig. S9–11. Additionally, the recovered powder of NSs from reactions employing octylamine at 230 °C also exhibits phase-pure 1T-TaS_2_ (Fig. S12).

**Fig. 3:**
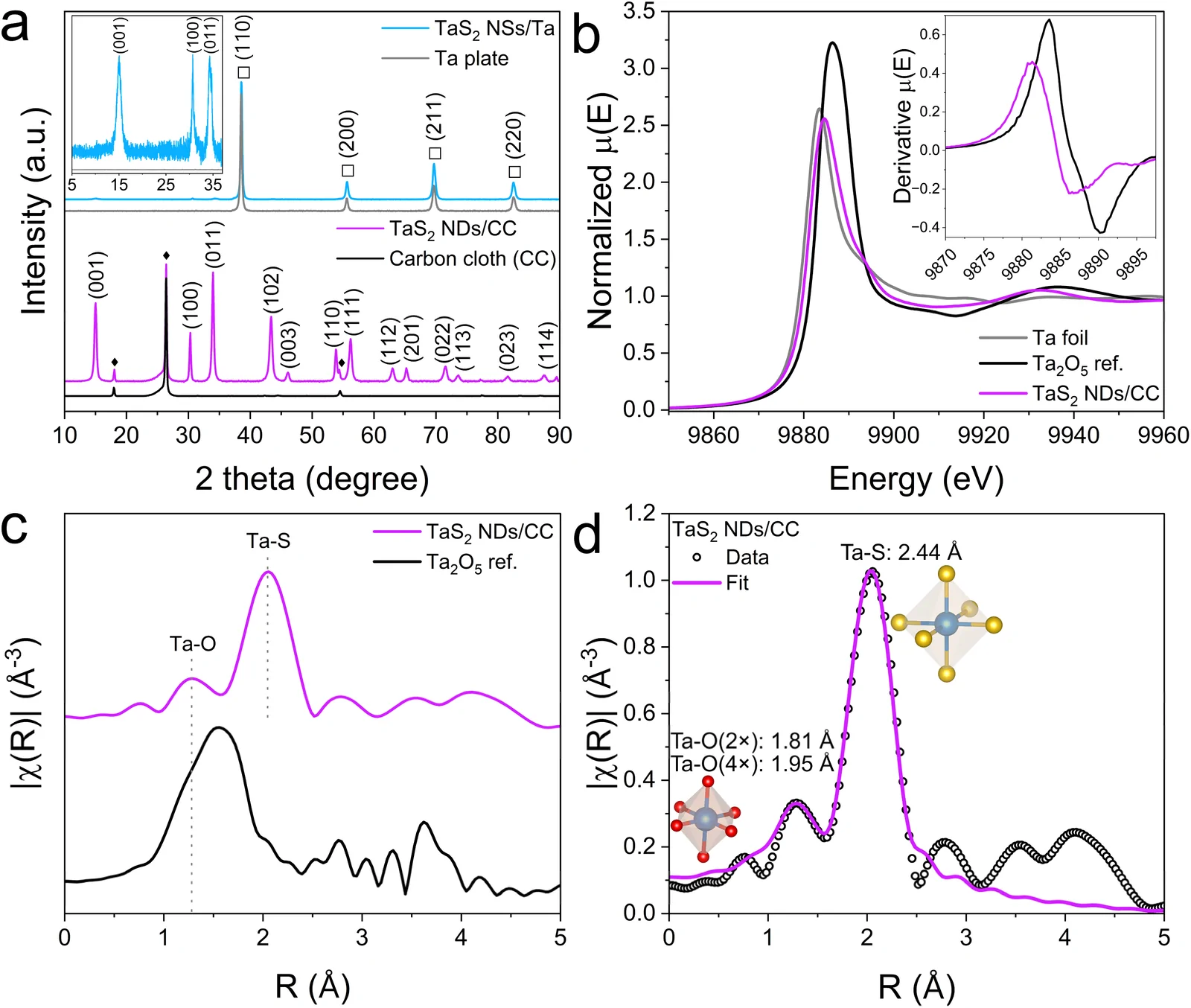
Structural and electronic characterization of TaS_2_ nanostructures (a) PXRD patterns of TaS_2_ NDs/CC and TaS_2_ NSs/Ta, with bare CC and Ta substrates shown for comparison. Symbols indicate reflections from metallic Ta (□) and CC (♦). Inset shows an enlarged view of the 2-theta region (5–37°) for TaS_2_ NSs/Ta. (b) Ta L_3_-edge XANES spectra of TaS_2_ NDs/CC, metallic Ta foil, and Ta_2_O_5_ reference, with the first-derivative spectra shown in the inset. (c) Corresponding *k*^2^-weighted Fourier-transformed EXAFS spectra of TaS_2_ NDs/CC and Ta_2_O_5_. Spectra are vertically offset. (d) EXAFS fit of TaS_2_ NDs/CC using the first coordination shell of TaS_2_ (Ta–S) and Ta_2_O_5_ (Ta–O). The inset shows the corresponding Ta-centered octahedral coordination environments and fitted coordination distances.

To investigate the oxidation state and local coordination environment of the TaS_2_ NDs directly grown on CC, we perform X-ray absorption spectroscopy (XAS) at the Ta L_3_-edge. Fig. 3b presents the X-ray absorption near-edge structure (XANES) spectra of TaS_2_ NDs/CC, Ta_2_O_5,_ and Ta foil, as reference samples. The absorption edge position (*E*_0_) of TaS_2_ NDs/CC is observed at 9881.38 eV, which is lower than that of Ta_2_O_5_ (9883.45 eV), and is accompanied by a less intense white-line feature. These characteristics indicate a lower oxidation state of Ta in TaS_2_ than in Ta_2_O_5,_ and they are consistent with Ta atoms coordinated by sulfur rather than by oxygen, as sulfur is less electronegative than oxygen. To gain further insight into the local atomic structure, we carry out extended X-ray absorption fine structure (EXAFS) analysis. As shown in Fig. 3c, the Fourier-transformed EXAFS spectrum of TaS_2_ NDs/CC exhibits a dominant peak at approximately 2.05 Å, which is assigned to Ta–S. In addition, a weak feature is observed at around 1.27 Å in the same *R*-space region as the Ta–O scattering contribution in the Ta_2_O_5_ reference spectrum. Thus, the EXAFS fitting using both Ta–S and Ta–O coordination shells provides a good description of the experimental data, confirming the TaS_2_ local structure while revealing a minor Ta–O contribution from Ta_2_O_5_ (Fig. 3d). Details on the EXAFS fitting are provided in SI Notes S1, Fig. S13, and Table S1. A certain degree of oxidation of TaS_2_ is expected, as layered chalcogenides are susceptible to oxidation under ambient conditions.^23,37^ Nevertheless, the XANES and EXAFS results indicate that the local and electronic structures remain dominated by the 1T-TaS_2_ phase, suggesting that oxidation is limited and does not significantly affect the bulk structure.

### 2.2 HER performance

To explore the electrocatalytic properties of the TaS_2_ nanostructures directly grown on different substrates, we evaluate their HER performance in a 0.5 M H_2_SO_4_ electrolyte, as shown in Fig. 4. The polarization curves obtained from linear sweep voltammetry (LSV) measurements without iR compensation (Fig. 4a) reveal a significant increase in HER activity for the self-supported TaS_2_ electrodes compared to bare substrates, CC and Ta plate. Among the samples, the TaS_2_ NSs grown on the Ta plate display the lowest overpotential at 10 mA cm^−2^ (*η*_10_) of 377 mV, which is considerably better than that of TaS_2_ NDs grown on the same substrate (*η*_10_ = 579 mV). The TaS_2_ nanostructures grown on the CC exhibit similar overpotentials, with NSs showing an *η*_10_ of 578 mV and the NDs an *η*_10_ of 553 mV. Although the NDs grown on the CC exhibit a slightly lower overpotential than the NSs, their catalyst loading is more than 30 times higher (10.03 *vs.* 0.32 mg cm^−2^), indicating that only a small amount of the NSs is sufficient to achieve HER performance comparable to that of the much more heavily loaded NDs. To further illustrate this effect, we present the mass-normalized current of the TaS_2_ NDs and NSs grown on CC in Fig. S14, with the NSs showing higher mass-normalized currents than the NDs. We hypothesize that this behavior is associated with the crumpled NSs architecture, which may facilitate electrolyte access to the TaS_2_ surface and alter the exposure and accessibility of catalytically relevant sites. In particular, the highly crumpled morphology of the thin NSs may offer a higher density of exposed edge sites, which contain under-coordinated atoms and are more active towards HER,^9,13,38^ compared with the larger and more compact NDs, which do not present such crumpling and may therefore expose a larger fraction of basal planes. The denser arrangement of the NDs may also limit the accessibility of part of the catalyst to the electrolyte. Our findings therefore suggest that the morphological characteristics of the NSs play a key role in enhancing HER performance, with the Ta plate identified as the most suitable substrate to maximize this effect. For comparison, the corresponding iR-compensated LSV curves are shown in Fig. S15 and confirm the same overall performance trend. Fig. S16 and S17 present the HER performance of TaS_2_ nanostructures synthesized with different reaction times on the CC and Ta plate. Additionally, the HER performance of TaS_2_ NDs and NSs grown on GC reveals that GC is the least suitable substrate for achieving efficient electrocatalysts, as shown in Fig. S18. This behavior is accompanied by poor mechanical stability of the TaS_2_ film, regardless of its morphology, during HER measurements, with progressive detachment from the substrate (Fig. S19).

**Fig. 4:**
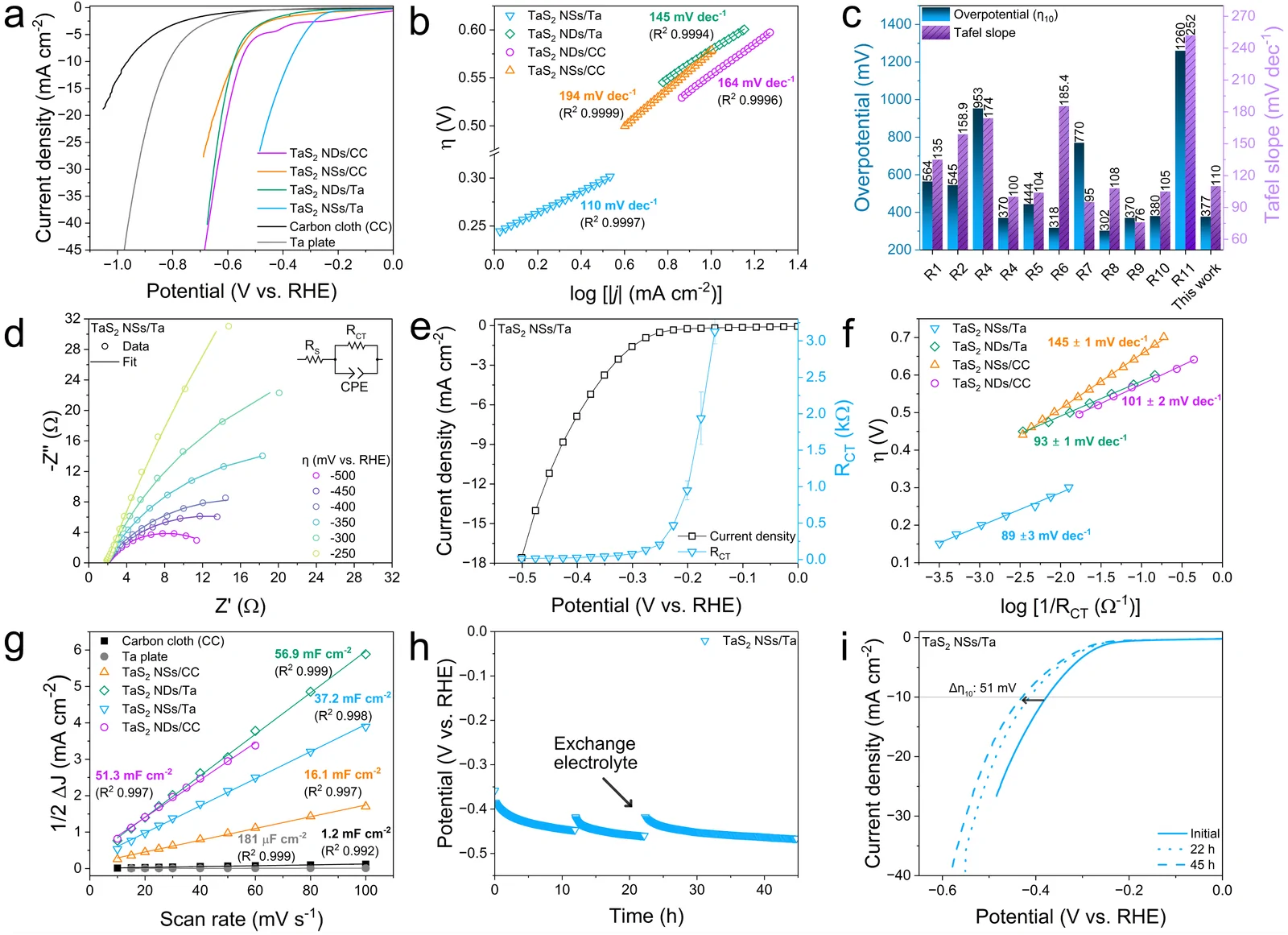
Evaluation of the HER performance of TaS_2_ nanostructures directly grown on substrates in 0.5 M H_2_SO_4_. (a) LSV curves of TaS_2_ NSs/Ta (230 °C, 12 h), TaS_2_ NDs/Ta (210 °C, 3 h), TaS_2_ NSs/CC (230 °C, 12 h), TaS_2_ NDs/CC (210 °C, 12 h), and bare CC and Ta substrates (without iR compensation). (b) Determination of the Tafel slopes from the curves in (a). (c) Comparison of the overpotential and Tafel slope of TaS_2_ NSs/Ta with literature-reported TaS_2_-based electrocatalysts in acidic media. R1–R11 denote the electrocatalysts listed in Table S2.^13,16,18,40–46^ (d) Nyquist plots from EIS measurements of TaS_2_ NSs/Ta at potentials near the HER onset. The inset shows the equivalent circuit used for fitting. (e) Charge-transfer resistance (*R*_CT_) obtained from fitting EIS data and current density as a function of applied potential for TaS_2_ NSs/Ta. (f) Tafel slopes derived from the EIS-fitted *R*_CT_ for the different TaS_2_ electrodes. (g) Determination of the double-layer capacitance (*C*_dl_) from linear fits of Δ*j*/2 as a function of scan rate; corresponding CV measurements are shown in Fig. S21. (h) Long-term stability of TaS_2_ NSs/Ta over 45 h at a constant current density of 10 mA cm^−2^, measured by chronopotentiometry. (i) LSV curves of TaS_2_ NSs/Ta before and after the stability test in (h).

In Fig. 4b, we determine the corresponding Tafel slopes. The TaS_2_ NSs on the Ta plate exhibit the lowest slope of 110 mV dec^−1^, while TaS_2_ NDs/Ta, TaS_2_ NDs/CC, and TaS_2_ NSs/CC show slopes of 145, 164, and 194 mV dec^−1^, respectively. The lower Tafel slope for the TaS_2_ NSs/Ta reveals more favorable HER kinetics compared to the other electrocatalysts. Moreover, a Tafel slope close to 120 mV dec^−1^ is generally associated with a Volmer-limited HER mechanism,^39^ in which the electrochemical adsorption of protons onto the catalyst surface to form adsorbed hydrogen constitutes the rate-determining step. The significantly higher values obtained for the other catalysts indicate slower HER kinetics. In the case of TaS_2_ NSs/CC, the higher Tafel slope compared with TaS_2_ NDs/CC, despite their similar overpotentials, may be related to the considerably lower catalyst loading of the NSs, which could result in a greater contribution from the underlying CC to the overall electrode response. However, these LSV-derived Tafel slopes should be interpreted with caution because they may be influenced by additional current contributions, such as capacitive charging and possible proton insertion processes, that are not directly associated with the HER. In general, we identify the TaS_2_ NSs on Ta plate as the most effective electrocatalyst for HER, exhibiting an overpotential lower than or comparable to that of some previously reported TaS_2_ nanostructures (Fig. 4c, Table S2). Notably, the present solvothermal synthesis yields a material with HER activity comparable to that of TaS_2_-based catalysts typically obtained through high-temperature solid-state reactions.

Additionally, we investigate the reaction kinetics using electrochemical impedance spectroscopy (EIS). Fig. 4d shows the Nyquist plots of TaS_2_ NSs grown on a Ta plate at selected potentials near the onset of the HER. As the potential becomes more cathodic, the impedance spectra evolve to a semicircular feature, indicating that the response is primarily governed by charge–transfer processes associated with the HER. The progressive reduction in the semicircle diameter indicates a decrease in the charge-transfer resistance (*R*_CT_), suggesting enhanced reaction kinetics at higher cathodic overpotentials. This behavior is further illustrated by the Nyquist plots obtained from EIS over the entire potential range (Fig. S20a) and is also observed for other TaS_2_ nanostructures grown on different substrates (Fig. S20c, e and g).

We fit the Nyquist plots using an equivalent circuit consisting of a series resistance (*R*_S_) in series with a parallel combination of *R*_CT_ and a constant phase element (CPE), as shown in the inset of Fig. 4d. Additional information on the EIS fitting is provided in SI Notes S2. Fig. 4e displays the evolution of the *R*_CT_ and the current density as a function of the applied potential. As the potential becomes more cathodic, the charge-transfer resistance decreases significantly while the current density increases due to the onset and progression of the HER, indicating enhanced electron-transfer kinetics at the electrode/electrolyte interface. For example, at a current density of *∼*11.2 mA cm^−2^, the fitted *R*_CT_ value is 19.1 Ω. All the TaS_2_ nanostructures on both CC and Ta exhibit a similar trend, as shown in Fig. S20d,f,h.

Using the *R*_CT_ values obtained from the EIS fitting, we calculate the Tafel slopes for all four electrocatalysts, as shown in Fig. 4f. The Tafel slopes derived from the EIS data are consistently lower than those obtained from the polarization curves in Fig. 4a and b. The TaS_2_ NSs and NDs grown on the Ta plate exhibit the lowest Tafel slopes of 89 and 93 mV dec^−1^, respectively, whereas the NSs and NDs grown on the CC show values of 145 and 101 mV dec^−1^, respectively. This behavior indicates more favorable kinetics than the polarization curves initially suggested. The discrepancy between the two methods may arise from additional contributions to the polarization curves, such as capacitive processes, that are not reflected in the *R*_CT_ values extracted from EIS. Nevertheless, the EIS-derived Tafel slopes do not alter the interpretation of the HER rate-determining step for the investigated electrocatalysts. Notably, the difference between the two methods is less pronounced for the TaS_2_ NSs grown on a Ta plate, the best-performing electrocatalyst, suggesting that its HER behavior is more closely governed by charge–transfer processes.

As shown in Fig. 4g, we determine the double-layer capacitances (*C*_dl_) of the self-supported TaS_2_ electrodes on different substrates by measuring cyclic voltammetry (CV) in the non-faradaic range (*cf.* Fig. S21). All samples exhibit higher *C*_dl_ values than their respective bare substrates, confirming the increased electrochemically accessible interface upon TaS_2_ growth. The TaS_2_ NDs display similar *C*_dl_ on both substrates (56.9 mF cm^−2^ on Ta plate *versus* 51.3 mF cm^−2^ on CC). In contrast, the TaS_2_ NSs display a lower *C*_dl_ value on the CC (16.1 mF cm^−2^) compared to the Ta plate (37.2 mF cm^−2^), which can be associated with the lower loading of NSs and reduced surface coverage of NSs on the CC substrate. Notably, the *C*_dl_ values do not directly correlate with the HER activity in Fig. 4a. Although *C*_dl_ is often used as an indicator of electrochemically active surface area, here it likely reflects contributions beyond the density of catalytically active sites, possibly related to the layered nature of TaS_2_. Moreover, the *C*_dl_ values for our self-supported TaS_2_ electrodes are consistent with those previously reported for TaS_2_ directly grown on various substrates.^15^

To evaluate the long-term durability of the self-supported TaS_2_ electrocatalysts, we employ prolonged chronopotentiometry measurements at a current density of 10 mA cm^−2^ (Fig. 4h and S22). Fig. 4h shows that TaS_2_ NSs grown on a Ta plate exhibit remarkable stability over ∼45 h, preserving HER activity with no significant fluctuations in current density. To confirm this observation, we compare the LSV measurements before the chronopotentiometry, after electrolyte exchange during the experiment, and at the end of the chronopotentiometry measurement, as depicted in Fig. 4i. After 22 h, following the electrolyte exchange, the overpotential increases from the initial value of 377 mV to 413 mV. After 45 h of chronopotentiometry, *η*_10_ reaches 428 mV, corresponding to an increase of only 51 mV relative to the initial value. Despite exhibiting overpotential values comparable to those of previously reported TaS_2_ nanostructures, the TaS_2_ NSs/Ta electrode displays stability over significantly longer testing periods than those investigated in earlier studies (see Table S2), highlighting the advantage of our electrode in terms of long-term durability. As evidenced in Fig. S22, the TaS_2_ NDs on the Ta plate, as well as both NDs and NSs on CC, exhibit similarly enhanced long-term stability, maintaining the HER activity for at least 47 h. For the TaS_2_ nanostructures grown on CC, the LSV curves before the long-term measurement, after electrolyte exchange, and at the end of the chronopotentiometry tests nearly overlap, indicating a negligible change in HER activity.

To investigate changes in the surface chemical composition of the electrocatalyst after the stability test (*i.e.*, *∼*45 h of operation), we employ X-ray photoelectron spectroscopy (XPS). In the Ta 4f core-level spectra (Fig. 5a), we observe an increase in the oxidation state of Ta in TaS_2_ NSs grown on a Ta plate after HER. The spectrum of the as-prepared TaS_2_ NSs exhibits three different contributions. The dominant doublet at 23.67 eV (4f_7/2_) and 25.58 eV (4f_5/2_) is assigned to Ta^4+^ in TaS_2_.^47,48^ At higher binding energies, the peaks at 26.51 eV (4f_7/2_) and 28.42 eV (4f_5/2_) correspond to Ta^5+^ in Ta_2_O_5_, in good agreement with previously reported values.^49–51^ The intermediate doublet at 24.77 eV (4f_7/2_) and 26.68 eV (4f_5/2_) suggests the presence of a reduced suboxide species (TaO_*x*_). Based on its binding energy shift relative to the fully oxidized state, this contribution is likely dominated by Ta^3+^;^51–53^ however, the presence of a species resulting from partial substitution of sulfur by oxygen (*e.g.*, TaS_2−*x*_O_*x*_) cannot be ruled out.^37^ The relative contributions of TaS_2_, TaO_*x*_, and Ta_2_O_5_ are ∼43.6 at%, ∼24.7 at%, and ∼31.7 at%, respectively. The presence of oxidized species on the material surface results from the tendency of these layered materials to oxidize upon exposure to ambient conditions.^23,37^

**Fig. 5:**
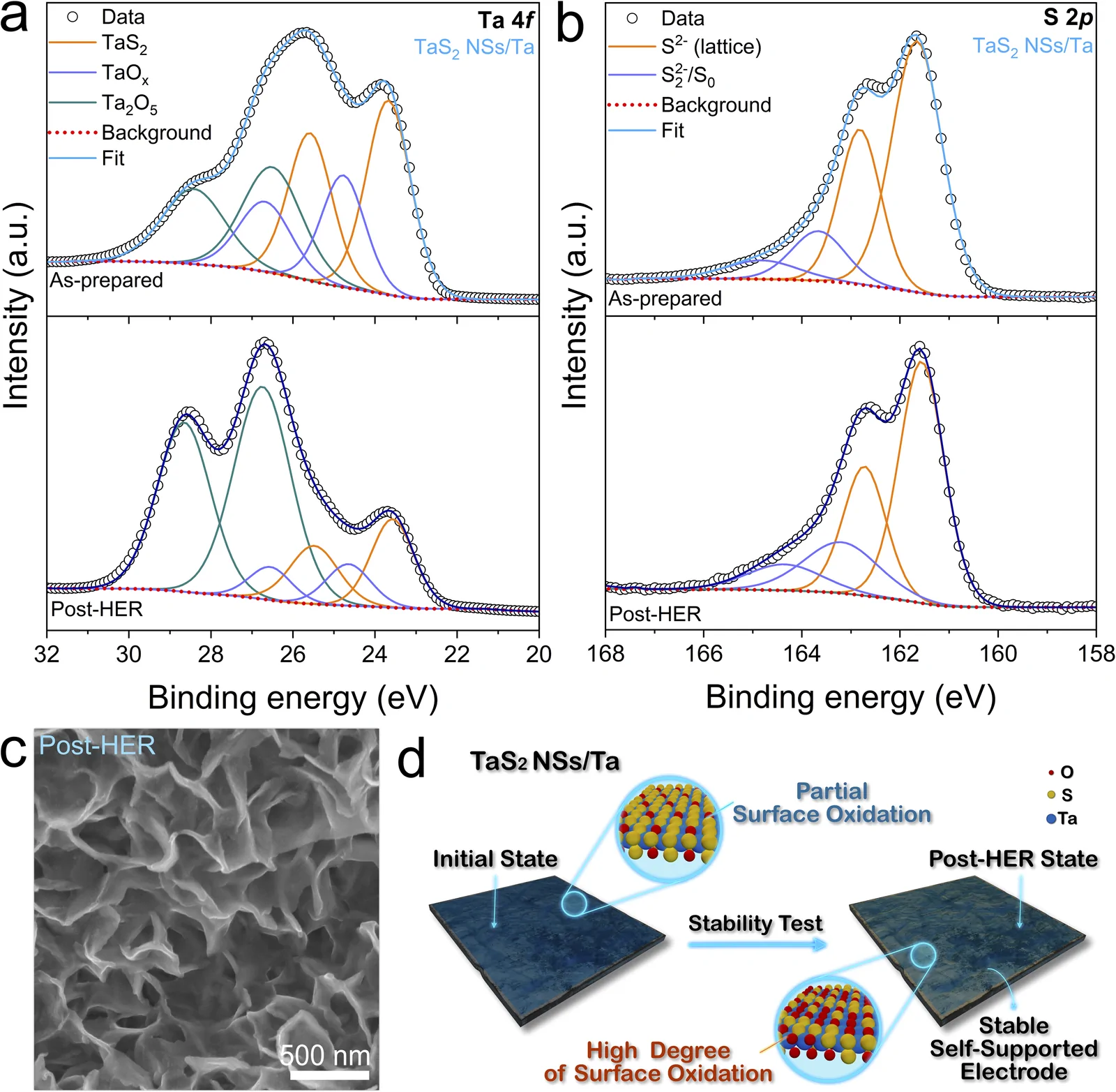
Post-HER characterization of TaS_2_ NSs grown on a Ta substrate. (a,b) Ta 4f and S 2p core-levels XPS spectra of TaS_2_ NSs/Ta, respectively, with fitted components, comparing the as-prepared and post-electrochemical samples. (c) SEM image of the TaS_2_ NSs/Ta after 45 h of HER operation, showing preservation of the original morphology. (d) Schematic illustration of the stability of the self-supported TaS_2_ NSs/Ta electrode after long-term HER operation.

After HER, the same three components are observed but with significantly different relative contributions. The Ta_2_O_5_ contribution (at 26.74 eV and 28.66 eV) becomes dominant (∼68.3 at%), whereas the TaS_2_ component (23.57 eV and 25.48 eV) decreases to ∼21.3 at%, and the TaO_*x*_ contribution (at 24.65 eV and 26.56 eV) to 10.4 at%. These results indicate extensive surface oxidation after the electrochemical measurements. However, the persistent TaS_2_ contribution suggests the sulfide phase remains on the electrocatalyst surface and may contribute to the retained HER activity. However, the *ex situ* XPS results do not unambiguously determine whether oxidation occurs entirely during exposure to the electrolyte and cathodic potentials or whether subsequent environmental exposure also contributes to the observed oxidation. Distinguishing between these two effects requires *in situ* or *operando* studies,^54^ which are beyond the scope of this work. Analysis of the XPS survey spectra further indicates an increase in oxygen content after HER (Fig. S23, Table S3).

Fig. 5b shows that the S 2p core-level spectra of both samples, before and after HER, exhibit similar features. The peaks at 161.56–161.65 eV (2p_3/2_) and 162.72–162.81 eV (2p_1/2_) are assigned to S^2−^ species in the lattice of 1T-TaS_2_. The persistence of the S^2−^ signal after prolonged HER further supports that the sulfide species associated with the TaS_2_ phase remain on the surface after electrocatalytic measurements. The secondary doublet at higher binding energy (2p_3/2_ at 163.19–163.66 eV and 2p_1/2_ at 164.35–164.82 eV) indicates sulfur in a different chemical environment, consistent with sulfur in a higher oxidation state relative to S^2−^, such as elemental sulfur or S_2_^−2^,^55^ possibly formed during partial oxidation of TaS_2_. An additional feature related to SO_4_^2−^ (169.33 eV and 170.49 eV) is seen after HER (*cf.* Fig. S23b). The O 1s core-level XPS spectra are shown in Fig. S23c.

As shown in Fig. 5c and S24, post-HER SEM images reveal that the wrinkled, sheet-like morphology of the TaS_2_ nanostructures on a Ta plate is preserved after ∼45 h of electrocatalytic operation. The TaS_2_ NSs remain interconnected across the surface of the Ta plate, indicating good structural stability of the self-supported electrode during HER operation. Only a slight increase in the thickness of the NSs is observed after the stability test, which may be associated with partial surface oxidation under electrochemical conditions. Therefore, we show that the direct growth of TaS_2_ nanostructures on substrates enables a structurally stable, self-supported electrode architecture that remains structurally intact during prolonged HER operation. The minor performance loss is mainly attributed to the partial surface oxidation of TaS_2_, an inherent characteristic of layered chalcogenide materials,^23,37^ rather than to mechanical degradation of the electrode (Fig. 5d). These results highlight the potential of the TaS_2_ NSs/Ta architecture as a robust and efficient electrocatalyst for sustained hydrogen production in acidic media.

## 3. Conclusions

In summary, we present a simple and effective strategy to overcome the challenges associated with the solution-phase synthesis of TaS_2_ nanostructures while enabling their direct growth on conductive substrates. This heterogeneous growth approach allows for the fabrication of self-supported electrodes composed of phase-pure 1T-TaS_2_. We demonstrate that both morphology control and substrate selection play key roles in determining the electrocatalytic performance. The TaS_2_ NSs directly grown on the Ta plate exhibit the highest HER activity and the most favorable HER kinetics, demonstrating that the interplay between catalyst morphology and substrate integration is crucial for optimizing HER performance. More importantly, all developed self-supported electrodes, whether TaS_2_ NDs or NSs grown on CC or Ta substrates, exhibit excellent long-term stability during HER operation for over 45 h, underscoring the advantages of the direct growth approach. Our findings highlight the potential of extending this growth strategy to other high-surface-area Ta-based substrates, such as foams, meshes, and felts. Overall, this work provides a versatile route for preparing TaS_2_ in both supported and powder forms, expanding opportunities for investigating TaS_2_ across a broad range of electrochemical applications.

## 4. Methods

### 4.1 Chemicals

Toluene anhydrous (99.8%), TaCl_5_ (99.995%), and octylamine (>99%) were provided by Thermo Scientific Alfa Aesar, while 1-dodecanethiol (1-DDT, ≥98%) and *tert*-dodecanethiol (*t*-DDT, 98.5%) were supplied by Sigma-Aldrich. Methanol (≥99.8%) and toluene (98%) for washing were acquired from VWR Chemicals and Sigma-Aldrich, respectively. For the production of the electrolyte, sulfuric acid (H_2_SO_4_, 96%) was obtained from Carl Roth. All chemicals were used as received without any further purification. The substrates, metallic Ta plate (15 mm × 15 mm × 0.5 mm), carbon cloth (*Ø* 20 mm, AvCarb P75T), and glassy carbon (15 mm × 15 mm × 0.5 mm) were purchased from Redoxme.

### 4.2 Synthesis of TaS_2_ nanodisks (NDs)

The solvothermal reactions were carried out in a glove box under an argon (Ar) atmosphere. In a typical synthesis, 0.5 mmol of TaCl_5_ was dissolved in 19.6 mL of anhydrous toluene and homogenized under magnetic stirring, forming an orange solution. Subsequently, 10 mmol of 1-DDT was added to the solution, which turned pale yellow, and the mixture was homogenized by further magnetic stirring. The solution was transferred to a Teflon liner containing the electrode substrate. For the Ta metal or glassy carbon plates, a Teflon holder was employed to keep the substrate in the vertical position (*cf.* Fig. S19a). The Teflon liner was sealed in the autoclave, which was heated in a conventional oven at 210 °C for different reaction times (3–36 h). After cooling to room temperature, the substrates containing the grown TaS_2_ were washed with toluene (5×), toluene and methanol 50 : 50 (3×), and methanol (5×), and left to dry under ambient conditions. The loading of the TaS_2_ nanostructures on CC was determined by weighing the substrate before and after the solvothermal reaction. Replacement of 1-DDT with *tert*-dodecanethiol (*t*-DDT) as the sulfur source under otherwise identical conditions also yielded crystalline TaS_2_ particles (Fig. S25).

### 4.3 Synthesis of TaS_2_ nanosheets (NSs)

A similar synthetic procedure was employed to control the morphology of TaS_2_. In the glove box under an Ar atmosphere, 0.5 mmol of TaCl_5_ was dissolved in 19.6 mL of anhydrous toluene and stirred magnetically, forming an orange solution. After dissolving TaCl_5_ in toluene, 2.5 mmol of octylamine was added to the solution, resulting in a transparent solution. Subsequently, 10 mmol of 1-DDT was added to the solution, which turned pale yellow, and the mixture was homogenized by further magnetic stirring. The solution was transferred to a Teflon liner containing the electrode substrate, as described before. The Teflon liner was sealed in the autoclave, which was heated in a conventional oven at 230 °C for different reaction times (3–36 h). After cooling to room temperature, the substrates containing the grown TaS_2_ were washed with pure toluene (5×), toluene and methanol (50 : 50, 3×), and pure methanol (5×), and left to dry under ambient conditions. If any powder was recovered in the synthesis, it was washed using the same conditions.

### 4.4 Characterization

#### 4.4.1 SEM

Scanning electron microscopy (SEM) and scanning transmission electron microscopy (STEM) images were obtained with a Regulus 8220 microscope (Hitachi High Technologies Corp., Japan) at 5 kV and 30 kV, respectively.

#### 4.4.2 PXRD

Powder X-ray diffraction (PXRD) patterns were collected on a Bruker D8 Advance diffractometer (Bruker Corp., USA) using a Cu Kα X-ray source (*λ* = 1.5418 Å), operated at 40 kV and 40 mA.

#### 4.4.3 TEM

Transmission electron microscopy (TEM), high-resolution TEM (HRTEM), STEM, and selected area electron diffraction (SAED) analyses were performed using a field-emission gun (FEG) Talos F200X microscope (Thermo Scientific/FEI, USA), operated at an acceleration voltage of 200 kV and equipped with a Super-X silicon-drift detector (SDD) system for energy-dispersive X-ray spectroscopy (EDX).

#### 4.4.4 XPS

X-ray photoemission spectroscopy (XPS) measurements were carried out on an XPS spectrometer equipped with an Al Kα X-ray source (1486.6 eV) and a Phoibos 150 hemispherical energy analyzer at DESY NanoLab.^56^ Data analysis was conducted using CasaXPS software. For peak fitting, a Shirley background was applied, and a mixed Gaussian–Lorentzian line shape (GL30) was used. The area ratio of the Ta 4f_7/2_ and 4f_5/2_ peaks was fixed at 4 : 3, while the area ratio of the S 2p_3/2_ and 2p_1/2_ peaks was fixed at 2 : 1.

#### 4.4.5 XAS

X-ray absorption spectroscopy (XAS) measurements were carried out at beamline P64 at PETRA III, Deutsches Elektronen-Synchrotron (DESY), Hamburg, Germany.^57^ Using a Si(111) monochromator, XAS data were collected at the Ta L_3_-edge (9881 eV) in transmission mode over an energy range of 9683–10683 eV. A Ta foil was used as a reference for energy calibration. *E*_0_ was defined as the maximum of the first derivative of µ(*E*) in the XAS spectra. The EXAFS data were processed and analyzed using the ATHENA and ARTHEMIS codes with the IFEFFIT software package.^58^ The phase shifts and amplitudes of the scattering paths were calculated using FEFF 6.0.^59^ The EXAFS spectra were Fourier transformed (FT) in the *k*-range from 3 to 12 Å^−1^ (Δ*k* = 9 Å^−1^) using a Hanning window with a *k*^2^ weighting. The bond lengths (*R*) and Debye–Waller factors (*σ*^2^) were fitted, while the many-body amplitude reduction factor (*S*_0_^2^) and energy shift (Δ*E*_0_) were fixed at 0.96 and 4.41 eV, respectively, as determined from fitting the Ta foil.

### 4.5 Electrochemical measurements

The self-supported electrodes were tested for electrochemical measurements without further treatment. The geometric surface area of each sample was determined by measuring the area in contact with the electrolyte. The measurements were carried out at room temperature in 0.5 M H_2_SO_4_ using a three-electrode cell, with an Ag/AgCl (3 M KCl) electrode as the reference electrode and a graphite rod as the counter electrode. The H_2_SO_4_ electrolyte was purged with Ar for 30 minutes before the electrochemical measurements. The data were collected using a Biologic SP-150 potentiostat. The linear sweep voltammetry (LSV) data were collected at a scan rate of 0.5 mV s^−1^, and the data were not compensated for the iR-drop. The double-layer capacitance (*C*_dl_) was determined from the cyclic voltammetry (CV) measurements recorded in a non-faradaic potential region at different scan rates between 10 and 100 mV s^−1^. Electrochemical impedance spectroscopy (EIS) measurements were conducted over a frequency range from 100 kHz to 0.1 Hz, with a 30 s equilibration time between each potential step. The resulting spectra were fitted using EC-Lab software (Biologic). Long-term stability was assessed by chronopotentiometry, applying a constant current calculated from the electrode geometric area to achieve a current density of 10 mA cm^−2^. The potential shift of the Ag/AgCl electrode to the RHE scale was corrected according to the equation *E*_(RHE)_ = *E*_(Ag/AgCl)_ + 0.059 pH + 0.197.

## Author contributions

C. A. Z.: conceptualization, investigation, writing – original draft, validation, visualization, methodology, formal analysis. F. C: investigation, validation, writing – review and editing, formal analysis, visualization, methodology. P. M. U.: investigation, methodology, writing – review & editing. E. S.: investigation, methodology, validation, writing – review & editing, formal analysis. Y. P.: investigation. J. K. K.: investigation, writing – review & editing. H. N.: investigation, writing – review & editing. F. M. T.: writing – review & editing, resources, investigation. A. S.: writing – review & editing, investigation, resources, methodology. D. K.: resources, supervision, writing – review & editing, project administration, conceptualization, funding acquisition.

## Conflicts of interest

There are no conflicts to declare.

## Supplementary Material

TA-OLF-D6TA07594K-s001

## Data Availability

All data generated and analyzed in this article are availabe at University of Hamburg ZFDM Repository at https://doi.org/10.25592/uhhfdm.21974. Supplementary information (SI) is available. See DOI: https://doi.org/10.1039/d6ta07594k.
