# Supplementary material for: Direct solvothermal growth of morphology-controlled TaS2 on substrates enables stable self-supported hydrogen evolution electrocatalysts
Source: J Mater Chem A Mater. 2026 Oct 26. Online ahead of print. doi: 10.1039/d6ta07594k (PMC13728942; doi:10.1039/d6ta07594k)
Supplement: Supplementary file 1 [file TA-OLF-D6TA07594K-s001.pdf]

Supplementary Information (SI) for Journal of Materials Chemistry A.
This journal is © The Royal Society of Chemistry

SUPPLEMENTARY INFORMATION

Direct solvothermal growth of morphology-controlled TaS_2 on substrates enables stable self-supported hydrogen evolution electrocatalysts

Cecilia A. Zito,[1,2]* Francesco Caddeo,[1] Philipp M. Ulmen,[1,2] Emil Schürmann,[1] Yvonne Pieper,[3] Jagadesh Kopula Kesavan,[1,2] Heshmat Noei,[5] Francesca M. Toma,[3,4] Andreas Stierle,[5,6] Dorota Koziej[1,2]*

[1] University of Hamburg, Institute for Nanostructure and Solid-State Physics, Center for Hybrid Nanostructures, Luruper Chaussee 149, 22761 Hamburg, Germany

[2] The Hamburg Centre for Ultrafast Imaging, Hamburg, Germany

[3] Institute of Functional Materials for Sustainability, Helmholtz-Zentrum Hereon, Kantstrasse 55, 14513 Teltow, Germany

[4] Faculty of Mechanical and Civil Engineering, Helmut Schmidt University, Holstenhofweg 85, 22043 Hamburg, Germany

[5] Centre for X-Ray and Nano Science CXNS, Deutsches Elektronen-Synchrotron DESY, Notkestrasse 85, 22607 Hamburg, Germany;

[6] Institute for Nanostructure and Solid-State Physics, University of Hamburg, Hamburg, Germany

***Corresponding authors:** cecilia.zito@uni-hamburg.de; dorota.koziej@uni-hamburg.de

Morphological characterization of the TaS_2 nanostructures on different substrates

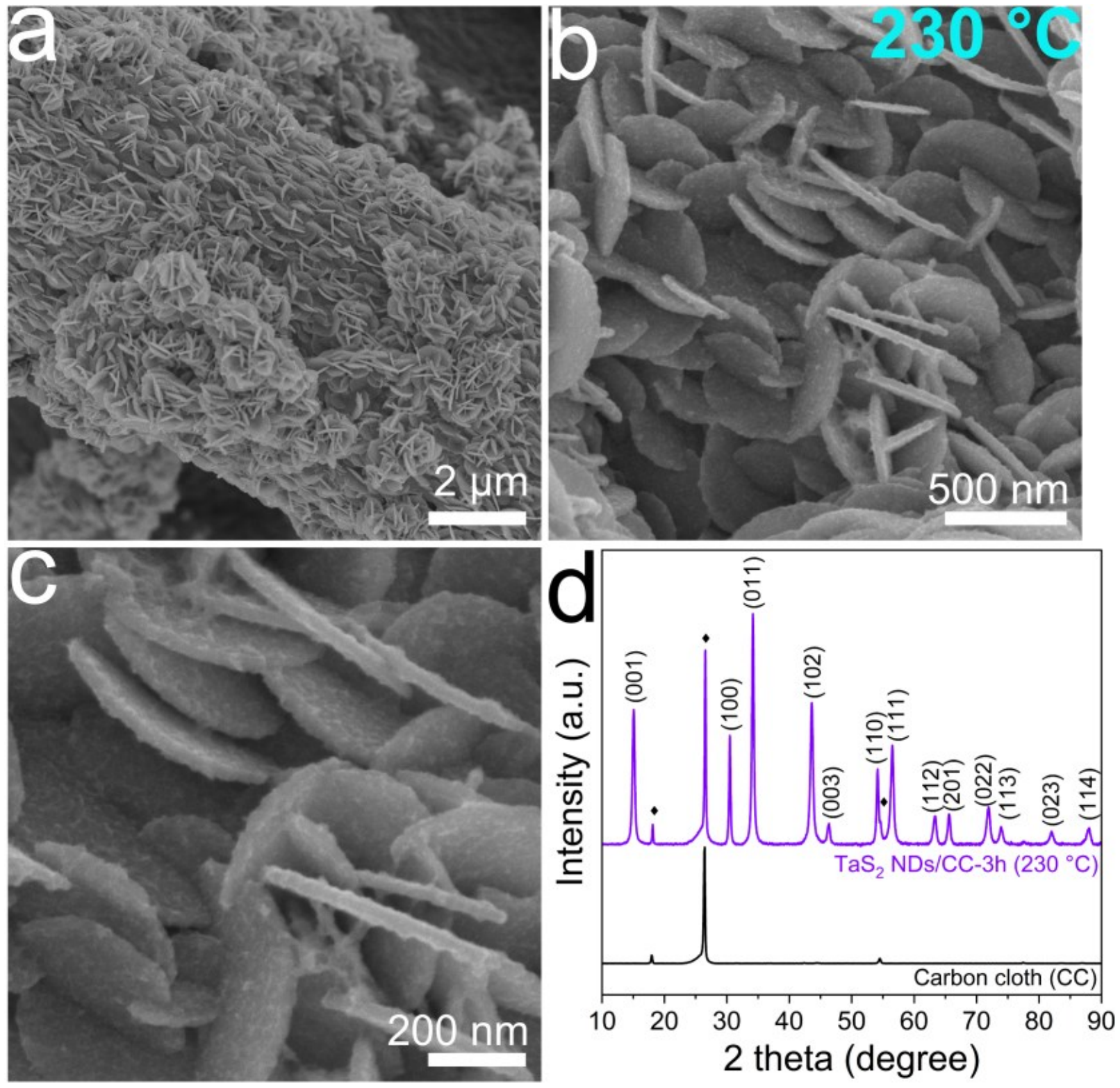

Figure S1. Characterization of TaS_2 nanodisks (NDs) grown on carbon cloth (CC) at 230 °C for 3 h in the absence of octylamine. (a-c) SEM images of TaS_2 at different magnifications, showing an average diameter and thickness of 513 ± 33 nm and 40 ± 7 nm, respectively. The characteristic morphology of the NDs is preserved when the temperature is increased from 210 to 230 °C. The TaS_2 loading was ~10.0 mg cm^{-2}, comparable to that obtained for the reaction carried out at 210 °C for 12 h. **(d)** PXRD pattern of the TaS_2 NDs/CC obtained at 230 °C for 3 h, where the symbol ♦ corresponds to CC reflections.

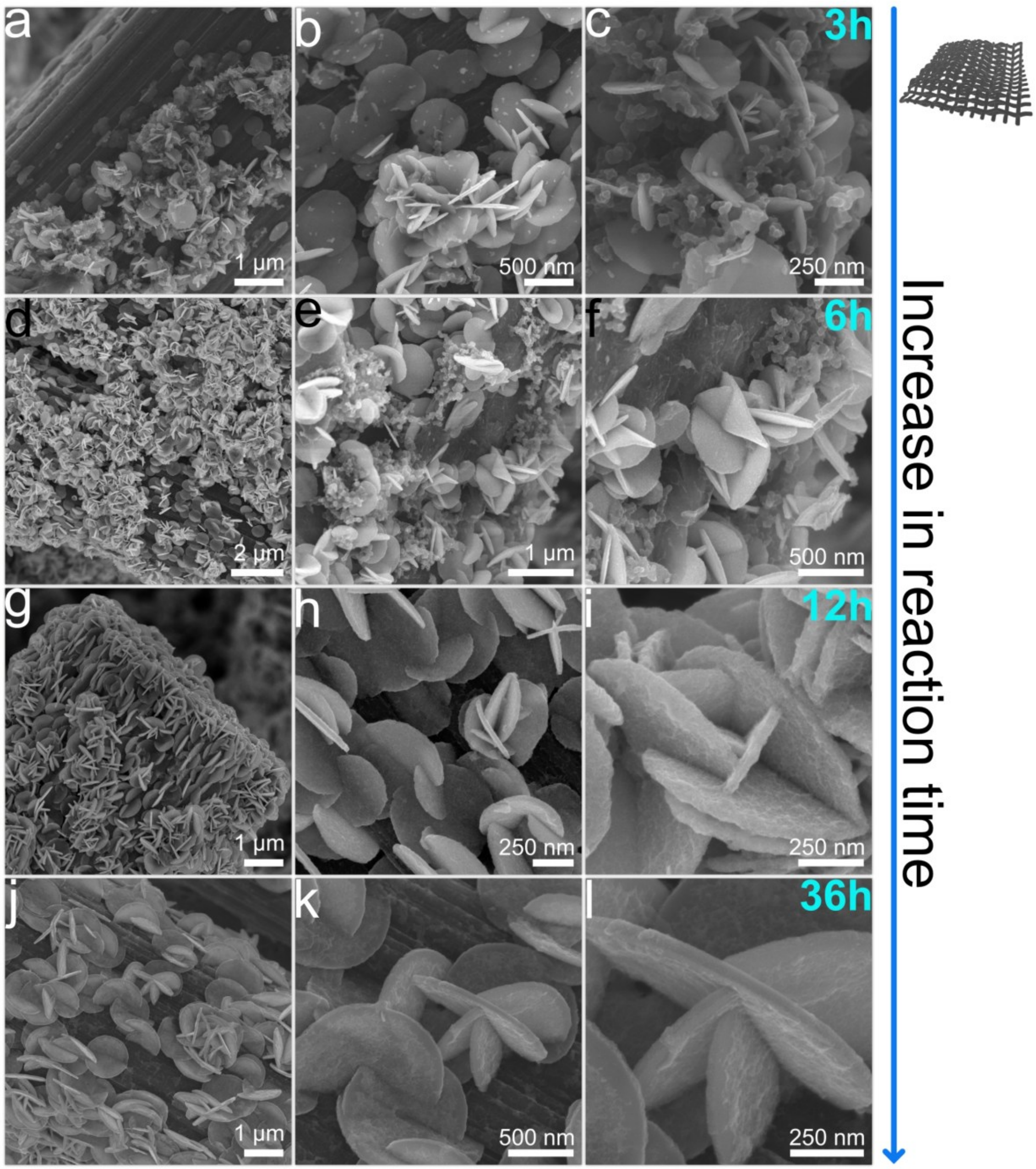

Figure S2. Morphological characterization of the TaS_2 nanodisks (NDs) grown on carbon cloth (CC) at 210 °C with different reaction times. SEM images of the TaS_2 NDs synthesized for **(a-c)** 3 h, **(d-f)** 6 h, **(g-i)** 12 h, and **(j-l)** 36 h. Approximate average diameters/thicknesses of 573 ± 96 nm/26 ± 6 nm, 442 ± 54 nm/34 ± 8 nm, 563 ± 72 nm/36 ± 8 nm, and 1074 ± 121 nm/52 ± 9 nm were estimated for reaction times of 3 h, 6 h, 12 h, and 36 h, respectively. The average TaS_2 loading on CC is approximately 1.91 mg cm^{-2}, 3.18 mg cm^{-2}, 10.03 mg cm^{-2}, and 17.3 mg cm^{-2} for reaction times of 3 h, 6 h, 12 h, and 36 h, respectively. The heterogeneous nature of the CC is evident in the images for the reaction times of 3 h and 6 h. In addition to the carbon fibers, small carbon particles embedded within the fibers and TaS_2 NDs are also visible, consistent with the particle-filled structure of the CC (Figure S3).

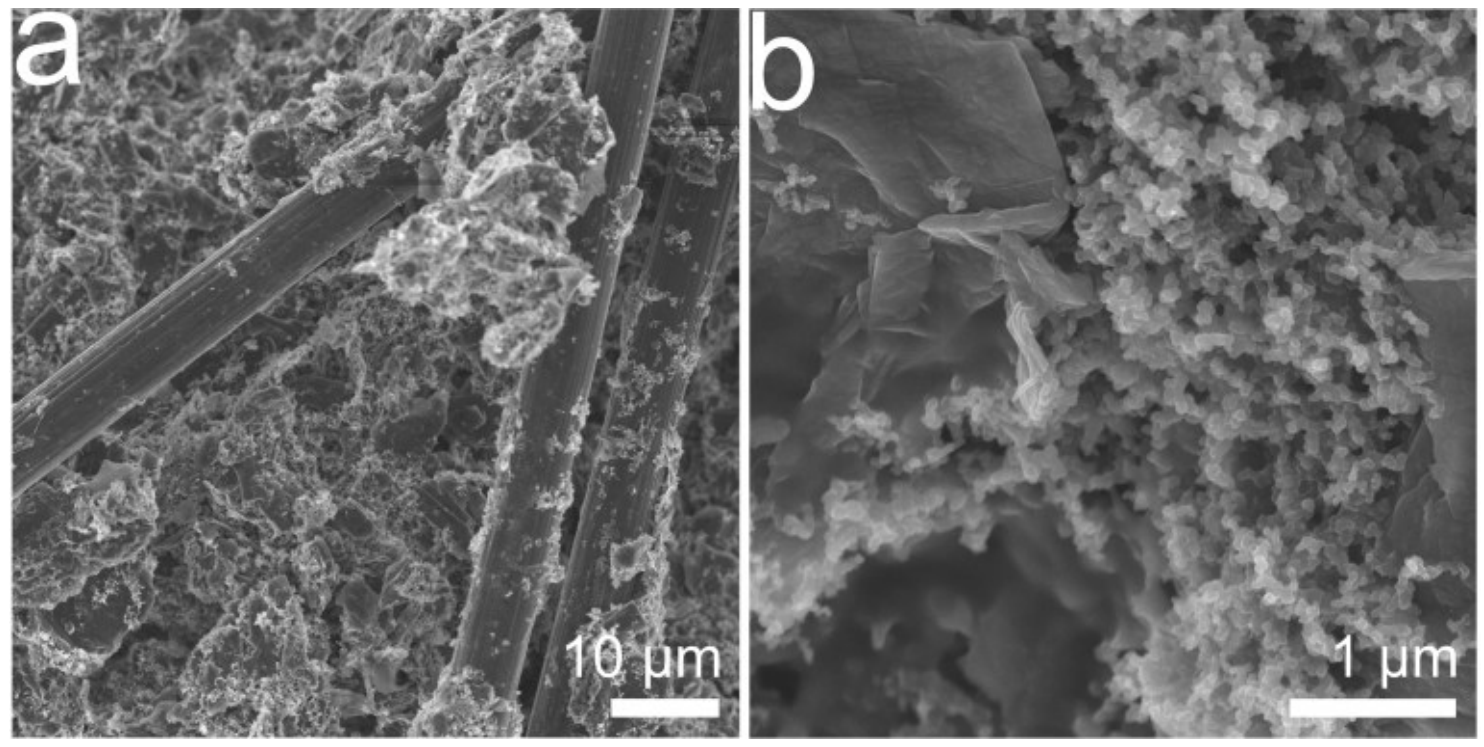

Figure S3. SEM images of the bare carbon cloth (CC). The CC is composed of carbon fibers, sheet-like structures, and small carbon particles embedded within the fibers.

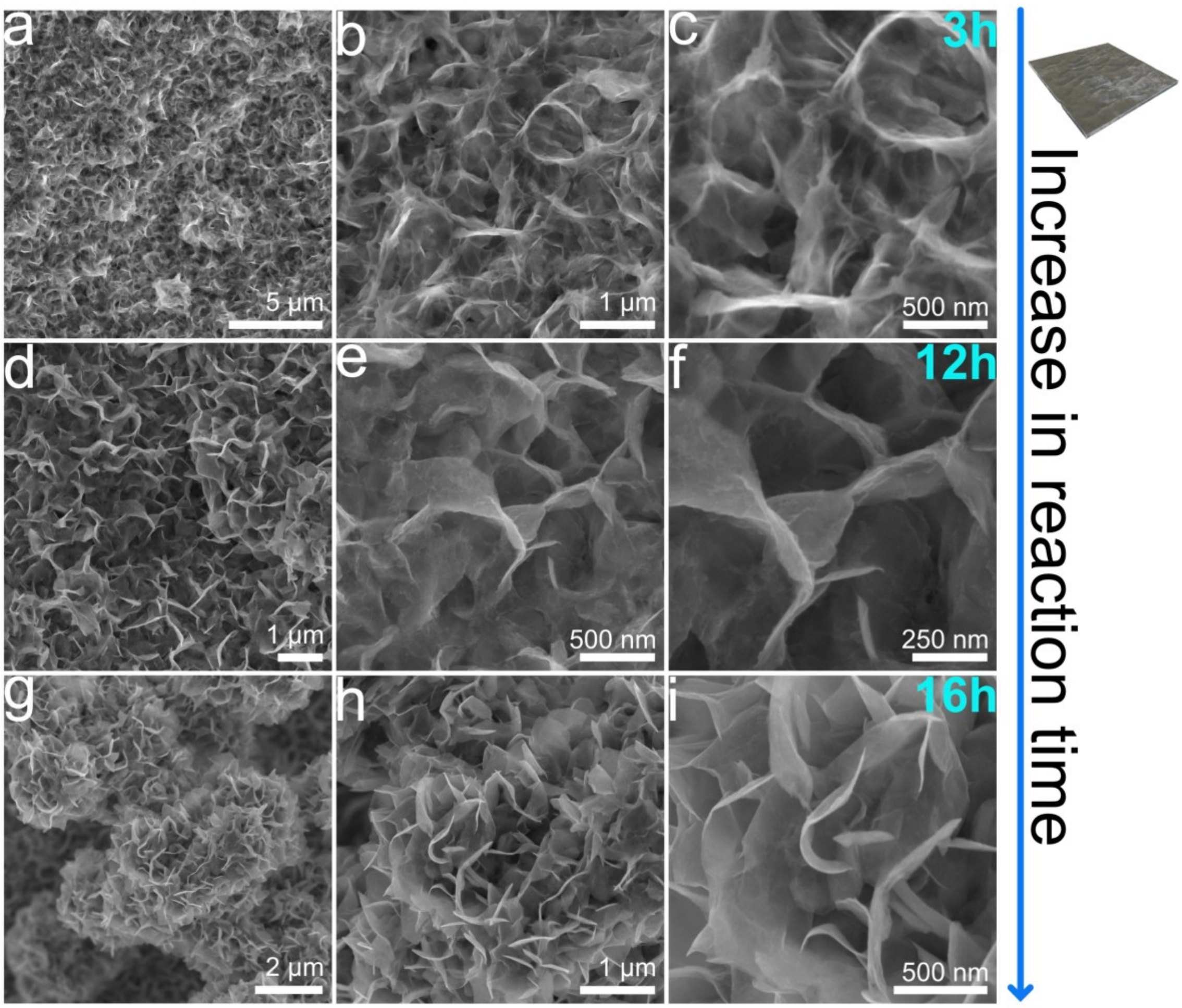

Figure S4. Morphological characterization of the TaS_2 nanosheets (NSs) grown on the Ta plate at 230 °C using octylamine with different reaction times. SEM images of the TaS_2 NSs synthesized after **(a-c)** 3 h, **(d-f)** 12 h, and **(g-i)** 16 h. The use of a short reaction time of 3 h yields thin interconnect nanosheets that are web-like. By increasing the reaction times to 12 h or 16 h, the NSs become more defined and crumpled.

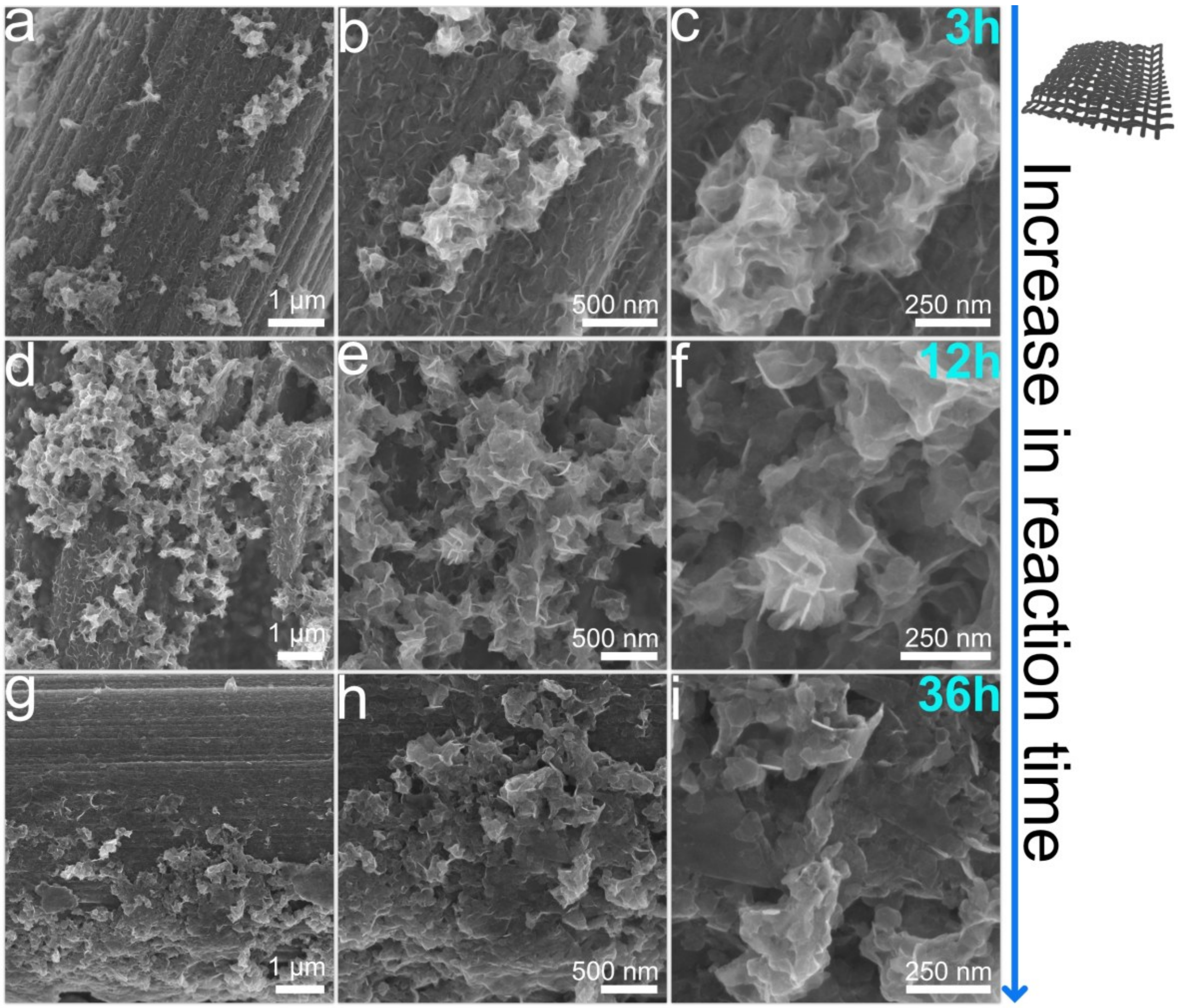

Figure S5. Morphological characterization of the TaS_2 nanosheets (NSs) grown on carbon cloth (CC) at 230 °C using octylamine with different reaction times. SEM images of the TaS_2 NSs synthesized after **(a-c)** 3 h, **(d-f)** 12 h, and **(g-i)** 36 h. The TaS_2 loading on the CC is approximately 0.32 mg cm^{-2} for all reaction times. The surface of the carbon fibers is covered by the thin NSs, which also form agglomerates on the surface. The reaction time of 36 h promotes the formation of a denser and thicker structure of the NSs.

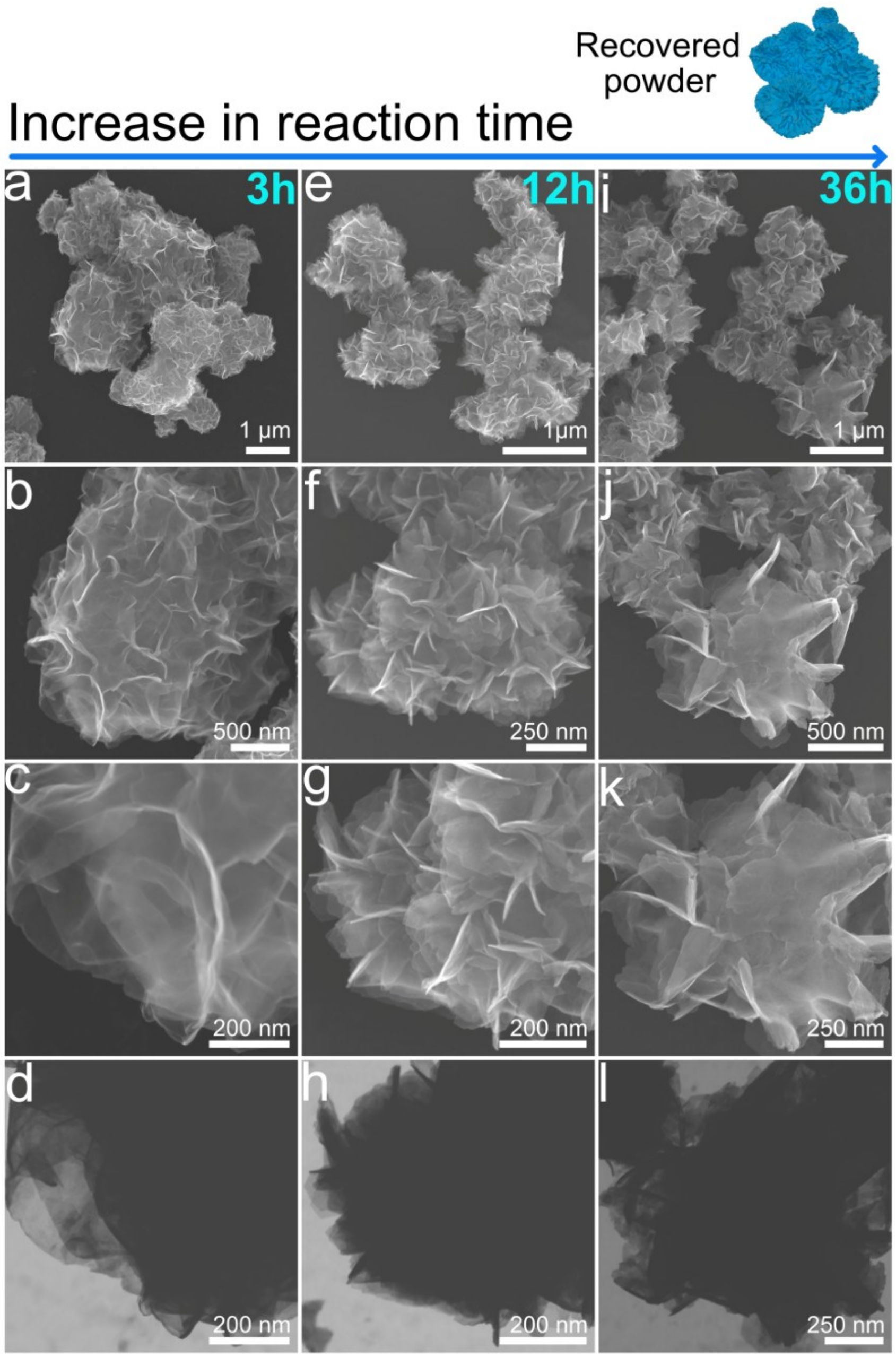

Figure S6. Morphological characterization of the TaS_2 powder recovered from reactions performed at 230 °C using octylamine and carbon cloth (CC) as the substrate for different reaction times. SEM images at different magnifications of the powders obtained after **(a-c)** 3 h, **(e-g)** 12 h, and **(i-k)** 36 h. The images reveal crumpled nanosheet morphologies for all reaction times. STEM images of the edges of the NSs of the samples synthesized for **(d)** 3 h, **(h)** 12 h, and **(l)** 36 h, showing their thin structure.

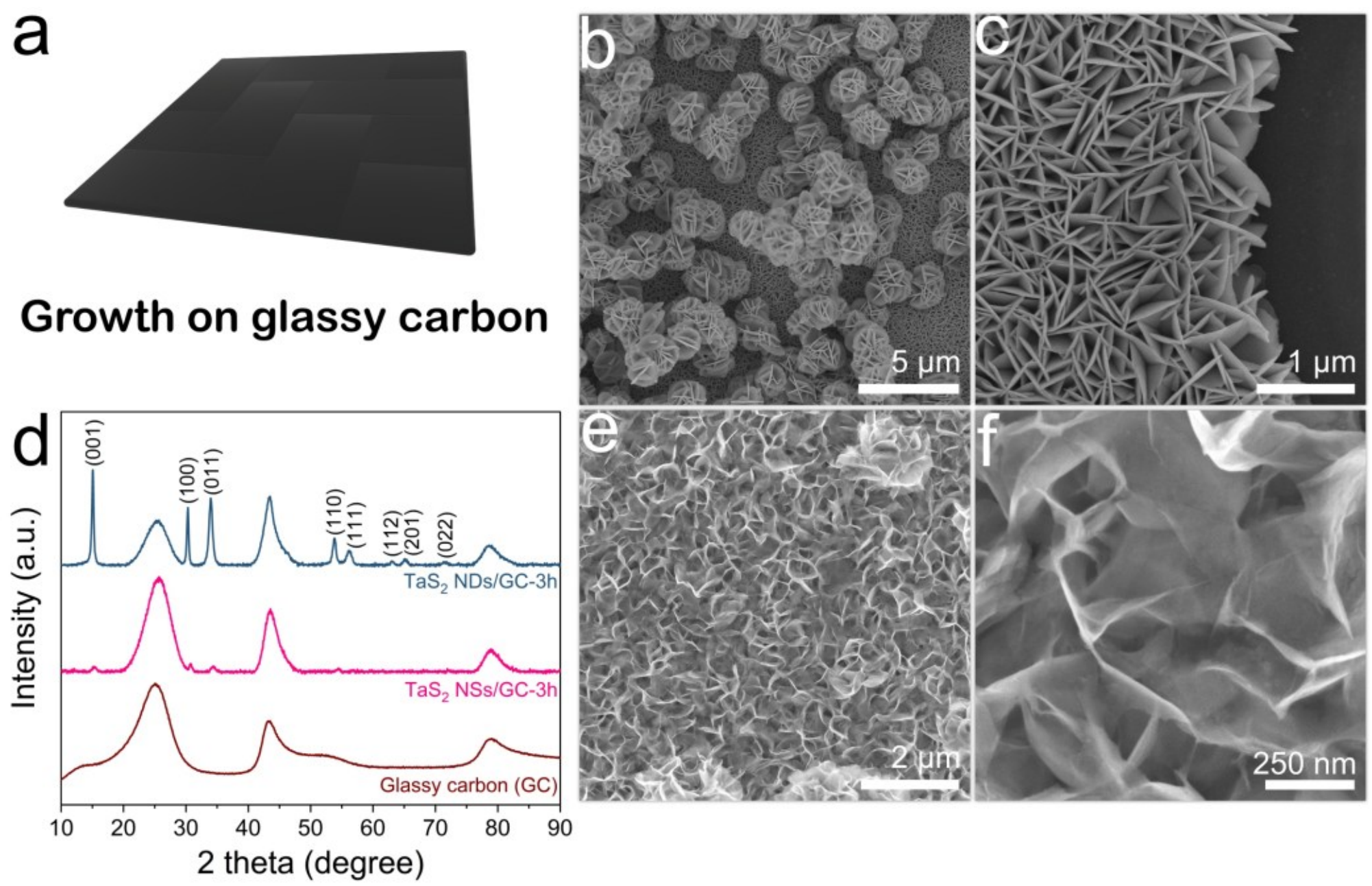

Figure S7. Characterization of the TaS_2 nanostructures grown on glassy carbon (GC). (a) Schematic illustration of the glassy carbon. **(b-c)** SEM images of the TaS_2 NDs synthesized at 210 °C for 3 h. **(d)** PXRD patterns of the TaS_2 nanostructures compared with bare GC. **(e-f)** SEM images of the TaS_2 NSs synthesized at 230 °C for 3 h with the addition of octylamine.

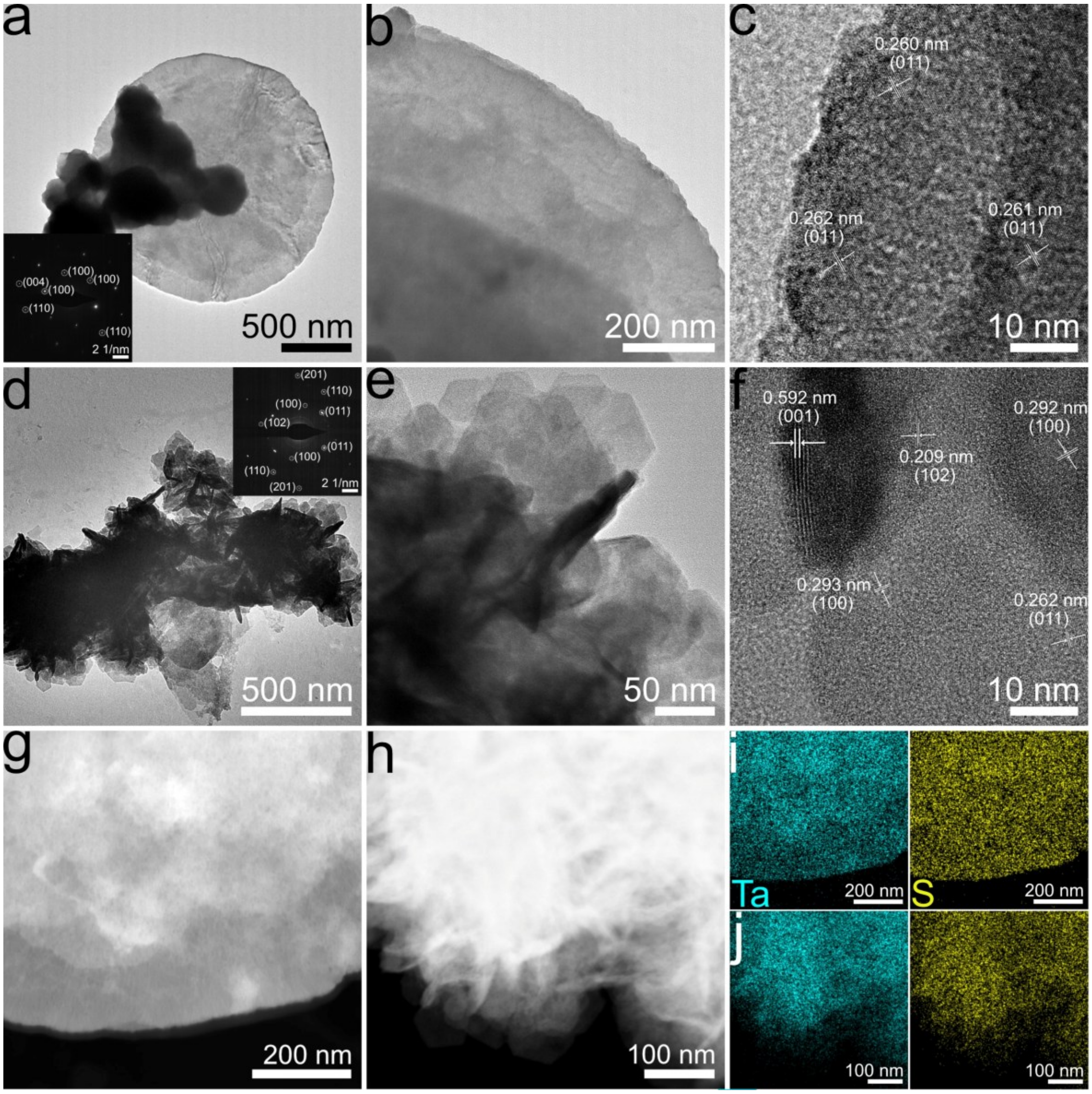

Figure S8. Morphology and structural characterization of TaS_2 nanostructures grown on a carbon cloth (CC). **(a-c)** TaS_2 NDs synthesized at 210 °C for 36 h: **(a,b)** TEM images displaying a single disk with a diameter of ≈1.7 µm (inset in (a) shows the SAED pattern that exhibits diffraction spots assigned to the 1T-TaS_2 phase), and **(c)** HRTEM image, showing only the (011) plane of 1T-TaS_2. **(d-f)** TaS_2 NSs obtained at 230 °C for 36 h using octylamine: **(d,e)** TEM images exhibiting agglomerated NSs that seem thicker (~15 nm thick) than those seen at shorter reaction times (inset in (d) corresponds to the SAED pattern that agrees well with the diffraction of 1T-TaS_2 phase), **(f)** HRTEM image, displaying the (001) interlayer spacing, as well as the (011) and (100) planes of 1T-TaS_2. **(g)** HAADF-STEM image of TaS_2 NDs and **(i)** the corresponding EDX elemental mapping of Ta and S. **(h)** HAADF-STEM image of TaS_2 NSs and **(j)** the corresponding EDX elemental mapping of Ta and S. Both elemental mappings show a homogeneous distribution of Ta and S throughout the sample.

Structural characterization of TaS_2 on different substrates

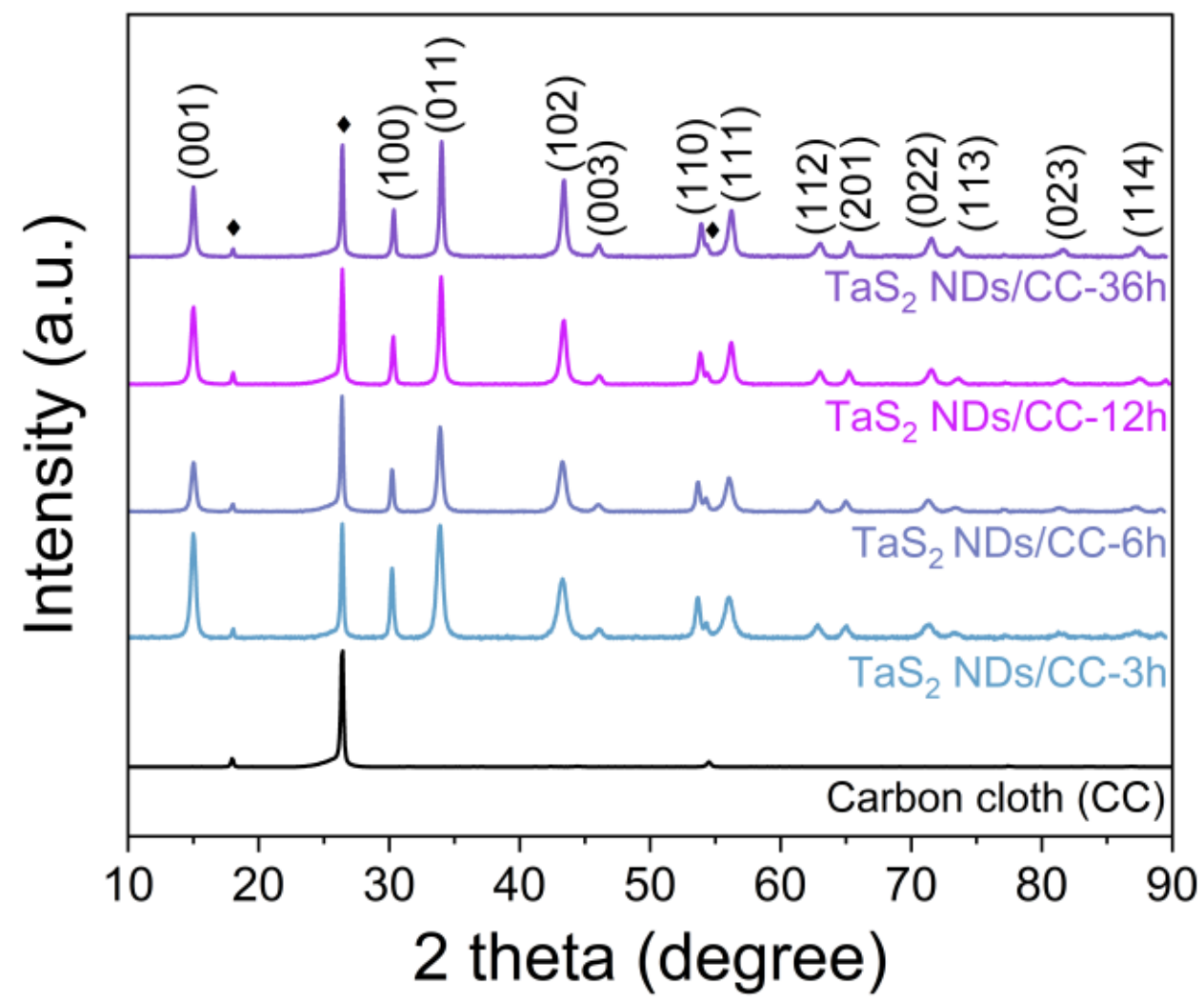

Figure S9. PXRD patterns of the TaS_2 nanodisks (NDs) directly grown on the carbon cloth (CC) substrate using different reaction times. The symbol ♦ indicates the reflections from the CC. The samples were synthesized at 210 °C without adding octylamine. The samples have a high degree of crystallinity even when synthesized using short reaction times such as 3 h. Crystallite dimensions were determined using the Scherrer equation from the (001) and (100) reflections. The (001) reflection was used to estimate the coherent domain size along the c-axis (layer-stacking direction of the TaS_2 layers), while the (100) reflection was used to evaluate the lateral coherent domain size within the ab plane. The values obtained from the (001) reflections were 16.6, 19.7, 19.6, and 24.4 nm for the TaS_2 NDs/CC synthesized for 3, 6, 12, and 36 h, respectively. These values can be used to estimate the number of layers within the crystallite domain using the equation $N_{layers} = {L_c}/{d_{001}}$, where N_{layers} is the approximate number of layers, L_c is the crystallite size obtained from (001) reflection, and d_{001} is the interlayer spacing (5.89 Å). Accordingly, the estimated number of layers is 28, 33, 33, and 41 for reaction times of 3, 6, 12, and 36 h, respectively, indicating progressive growth along the stacking direction. The lateral coherent domains estimated from the (100) reflection were 30.2, 32.7, 31.4, and 34.4 nm for reaction times of 3, 6, 12, and 36 h, respectively, showing a comparatively smaller increase in the lateral dimension than in the stacking direction.

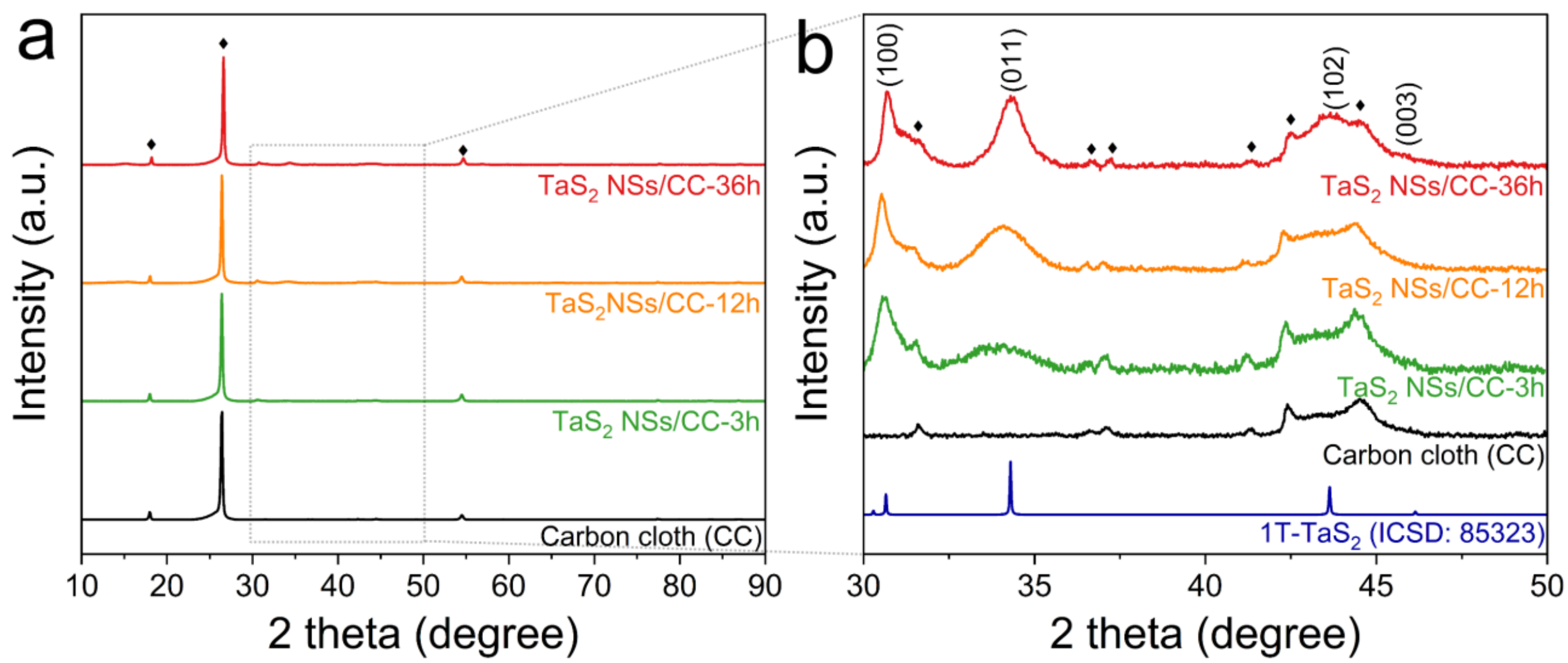

Figure S10. Effect of octylamine and reaction time on TaS_2 growth on carbon cloth (CC) as a substrate. (a) PXRD patterns of the TaS_2 nanosheets (NSs) directly grown on the carbon cloth (CC) substrate using different reaction times. The symbol ♦ indicates the reflections from the CC. The samples were synthesized at 230 °C with the addition of octylamine. **(b)** Enlargement of the region (30-50°) to evidence the reflections of TaS_2, confirming the loading of particles into the CC. The low intensities of the TaS_2 reflections are in line with the loading of only *ca*. 0.32 mg cm^{-2}.

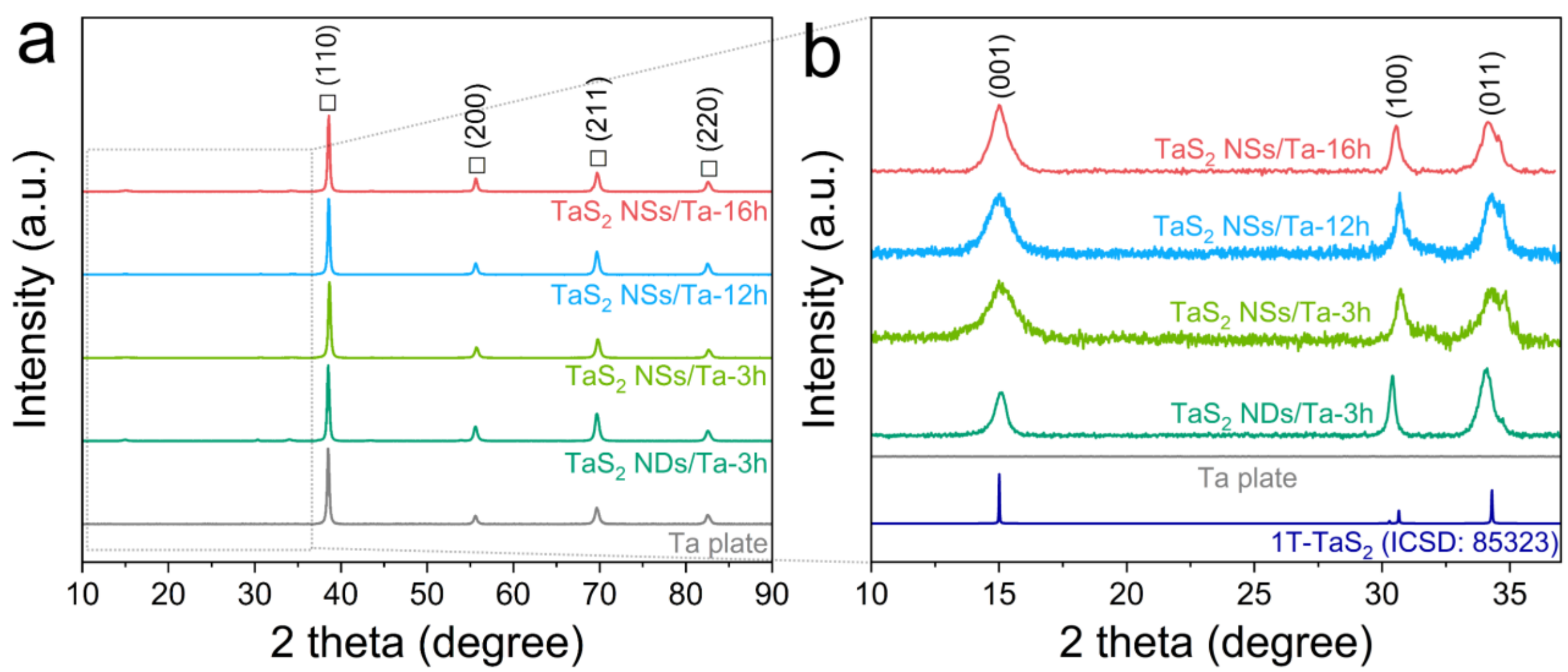

Figure S11. (a) PXRD patterns of the nanosheet-like TaS_2 directly grown on the Ta plate substrate using different reaction conditions. The symbol □ is indexed as the reflections from metallic Ta (cubic, Im-3n (229)). **(b)** Enlargement of the 2-theta region (10-37°) to evidence the reflections of TaS_2, confirming the growth of the TaS_2 film on the Ta plate. For the TaS_2 NSs/Ta, the coherence lengths estimated from the (001) reflection were 6.4, 7.5, and 9.1 nm for reaction times of 3, 12, and 16 h, respectively, corresponding to approximately 11, 13, and 15 stacked layers. The lateral coherent domain sizes from the (100) reflection ranged from 19.9 to 26.6 nm. For the TaS_2 NDs/Ta, the c-axis coherence length was 15.6 nm (26 layers), while the lateral coherent domain size was 32.2 nm.

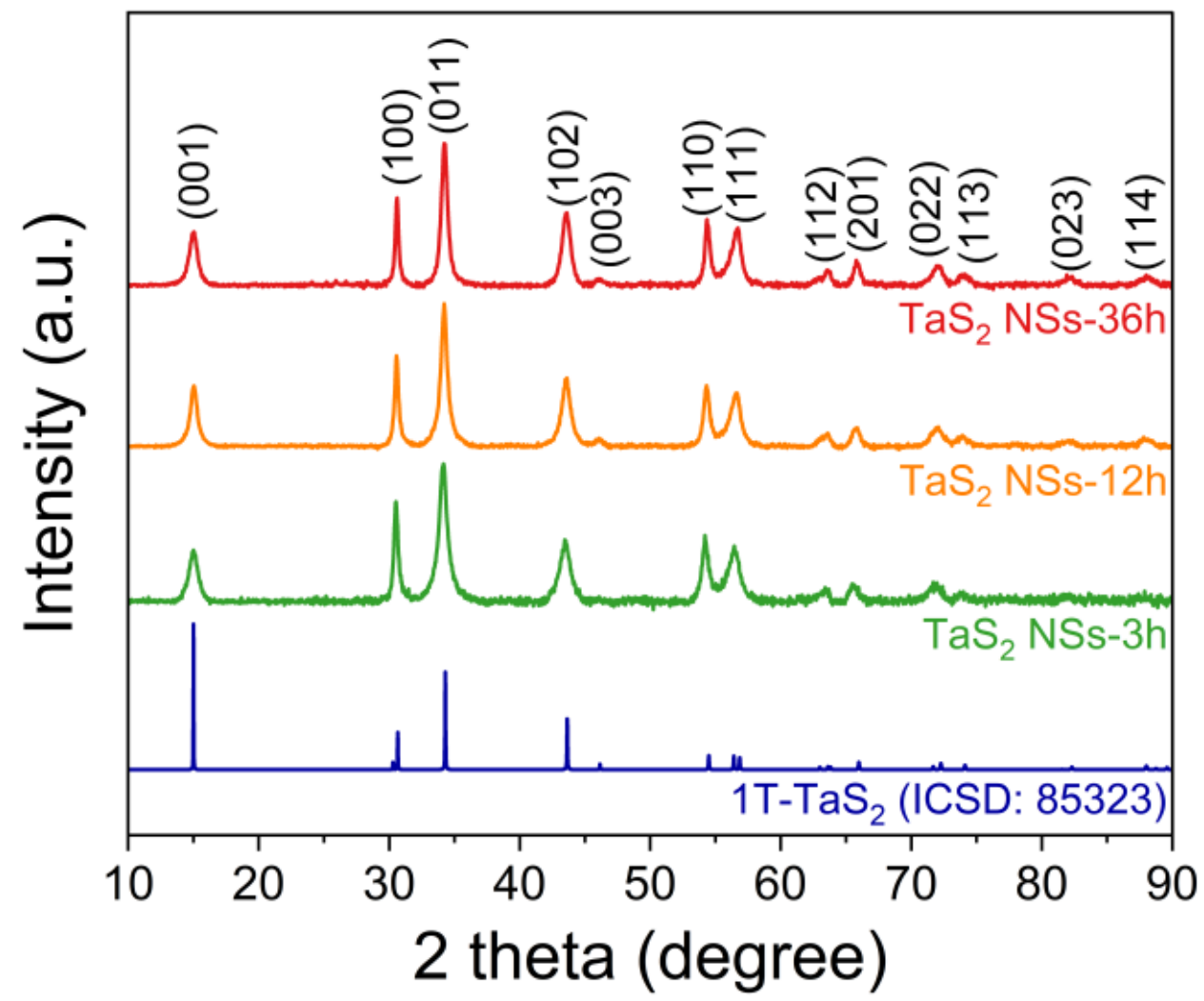

Figure S12. PXRD patterns of the recovered powder from the reactions using octylamine at 230 °C. By using CC, some powder can be collected from the reaction mixture, in addition to obtaining the self-supported electrode. For the TaS_2 NSs powder samples, the c-axis coherence lengths estimated from the (001) reflection were 10.1, 12.8, and 10.2 nm for reaction times of 3, 12, and 36 h, respectively, corresponding to approximately 17, 22, and 17 stacked layers. The lateral coherent domain sizes estimated from the (100) reflection were 22.6, 24.6, and 25.1 nm for the 3, 12, and 36 h samples, respectively.

Supplementary Notes 1: Extended X-ray absorption fine structure (EXAFS) analysis

We investigate the local coordination environment of Ta in the TaS_2 NDs grown on CC by fitting the Fourier-transformed EXAFS data in R-space, as shown in Figure 3d. The refinement using a model based only on the 1T-TaS_2 phase does not adequately reproduce the experimental spectrum, with noticeable discrepancies in the region R below 1.8 Å. The feature centered at 1.27 Å in the FT-EXAFS spectrum overlaps with the Ta-O scattering contribution from the Ta_2O_5 reference, indicating the presence of an oxide contribution (cf. Figure 3c). Therefore, an additional contribution from tantalum oxide is introduced into the fitting model.

To account for the coexistence of sulfide and oxide local environments, the scattering paths from the TaS_2 [1] and Ta_2O_5 [2] phases were fitted using a linear-combination model. The amplitudes of the TaS_2 and Ta_2O_5 contributions were scaled by $S_0^2 * x$ and $S_0^2 * (1-x)$, respectively, where x was refined during the fitting procedure. To minimize ambiguity arising from the substantial overlap of higher-shell scattering paths from the sulfide and oxide environments (e.g., Ta-Ta contributions), only the first-shell Ta-S and Ta-O single-scattering paths were included in the fit. Due to strong correlations among the fitting parameters, an iterative refinement strategy was employed.

The combined TaS_2/Ta_2O_5 model provides a good description of the experimental data, yielding an R-factor of 0.01118, indicating good agreement between the experimental and calculated spectra. The refined parameter x = 0.69(9) supports the inclusion of an oxide contribution to reproduce the EXAFS data. However, x should be regarded only as a weighting factor within the structural fitting model that reflects the relative contributions of the selected scattering amplitudes and not as a direct measure of the oxide content in the sample. This interpretation is consistent with the XANES results, which do not show a pronounced shift of the absorption edge toward higher energies, as expected for extensive oxidation to Ta^{5+}. Therefore, although EXAFS reveals the presence of a population of Ta atoms in an oxygen-coordinated environment, both EXAFS and XANES analyses indicate that the local and electronic structure remains dominated by TaS_2. The quantitative fitting parameters are summarized in **Table S1**, where the parameters for one Ta-O scattering path were fixed at the values obtained from the constrained refinement due to strong correlations with other fitting parameters. The experimental and fitted back-Fourier-transformed $k^2\chi(q)$ spectra are shown in **Figure S13**.

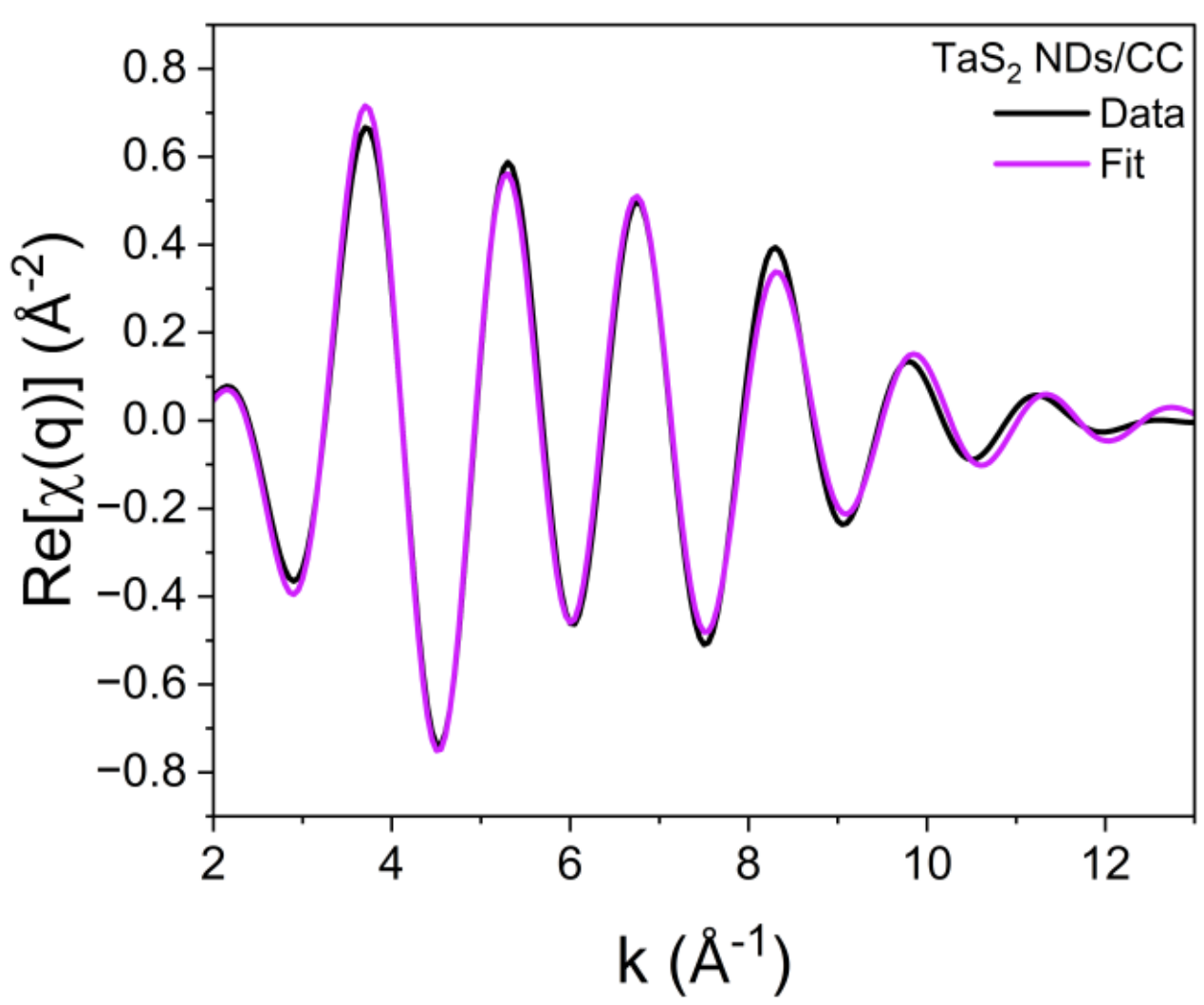

Figure S13. Experimental and fitted back-Fourier-transformed Ta L_3-edge $k^2\chi(q)$ spectra in reciprocal space of TaS_2 NDs/CC. The corresponding fitted data in R-space are shown in Figure 3d.

Table S1. EXAFS fitting parameters.

Scattering path	N	R (Å)	σ^2 (Å^2)
Ta-S	6	2.444(4)	0.0096(16)
Ta-O	2	1.813*	0.00503*
Ta-O	4	1.95(3)	0.0088(35)

N: coordination number, R: coordination distance, σ^2: Debye-Waller factor
* Fixed at the value obtained from the constrained sequential refinement

Electrocatalytic performance of TaS_2 nanostructures

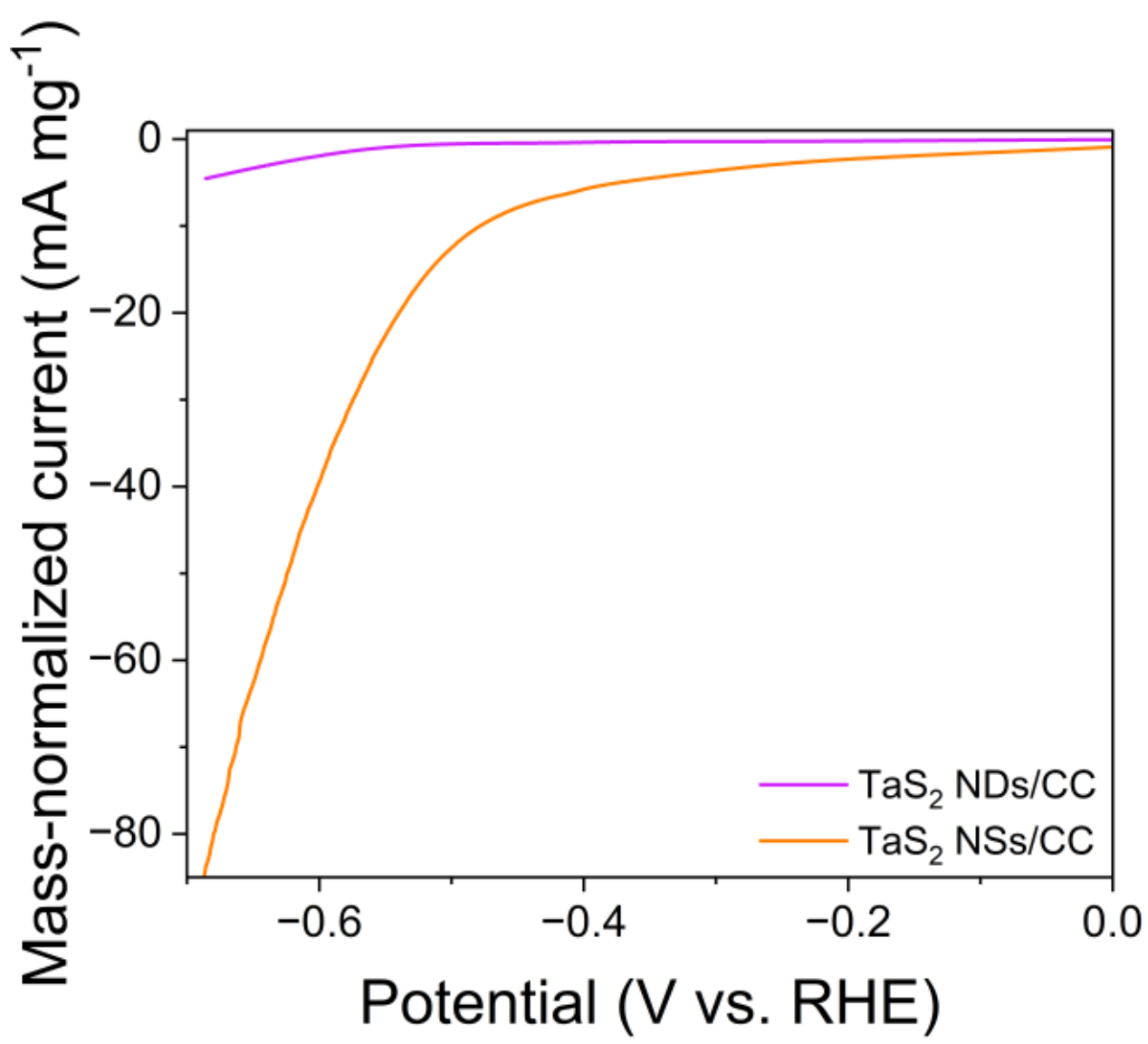

Figure S14. LSV curves of TaS_2 NDs/CC and TaS_2 NSs/CC with the current normalized by the TaS_2 mass loading. The NSs exhibit more than 18 times higher current than the NDs at the highest measured cathodic potential (*ca*. -0.69 V vs. RHE).

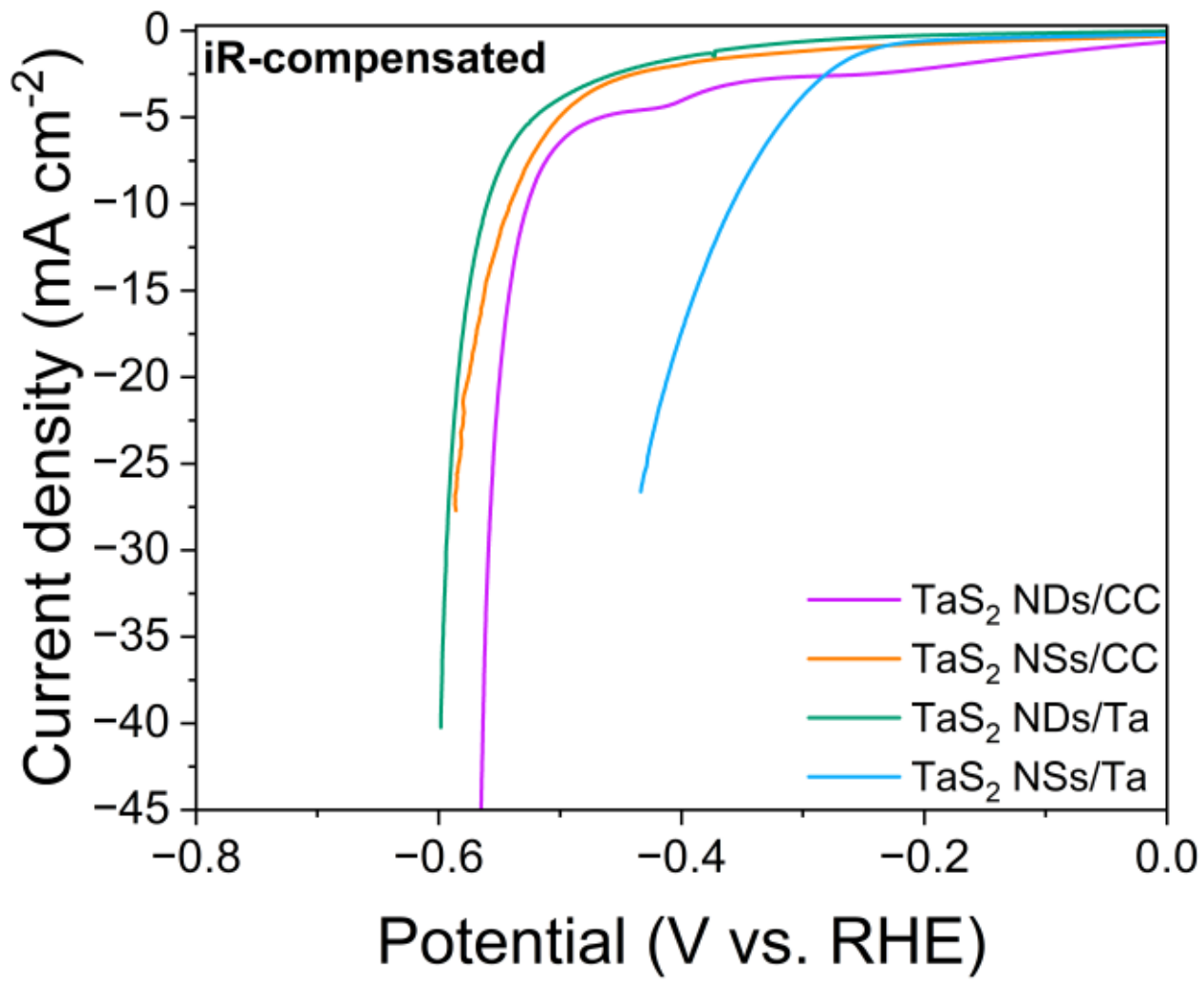

Figure S15. iR-compensated polarization curves corresponding to the LSV measurements shown in Figure 4a. The η_{10} values after iR compensation are 358, 560, 541, and 528 mV for TaS_2 NSs/Ta, TaS_2 NDs/Ta, TaS_2 NSs/CC, and TaS_2 NDs/CC, respectively.

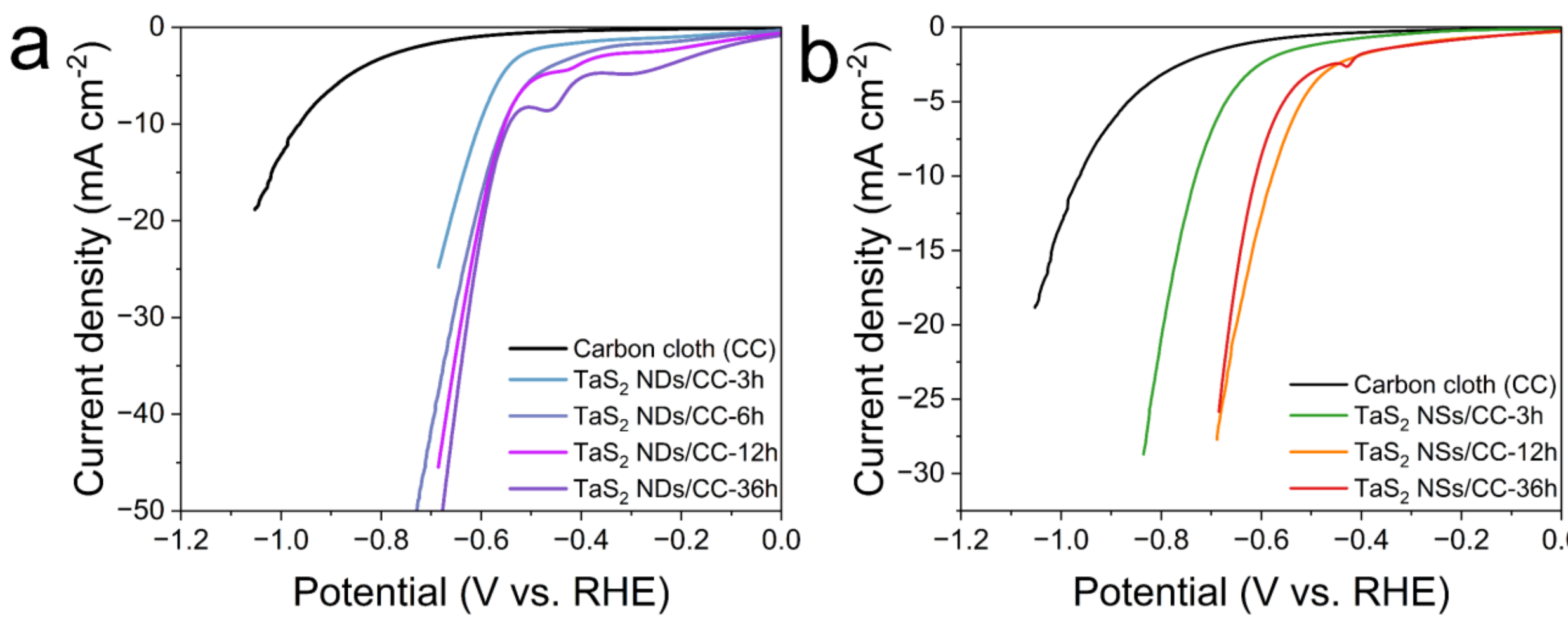

Figure S16. Effect of the reaction time on the HER activity of TaS_2 nanostructures grown on carbon cloth (CC). **(a)** Polarization curves of TaS_2 NDs grown on CC prepared with reaction times of 3-36 h. The η_{10} values are 603, 556, 553, and 544 mV for samples synthesized for 3, 6, 12, and 36 h, respectively, indicating minor variation in activity with reaction time. **(b)** Polarization curves of TaS_2 NSs grown on CC synthesized with reaction times of 3-36 h. The η_{10} values are 731, 578, and 610 mV for the samples synthesized for 3, 12, and 36 h, respectively.

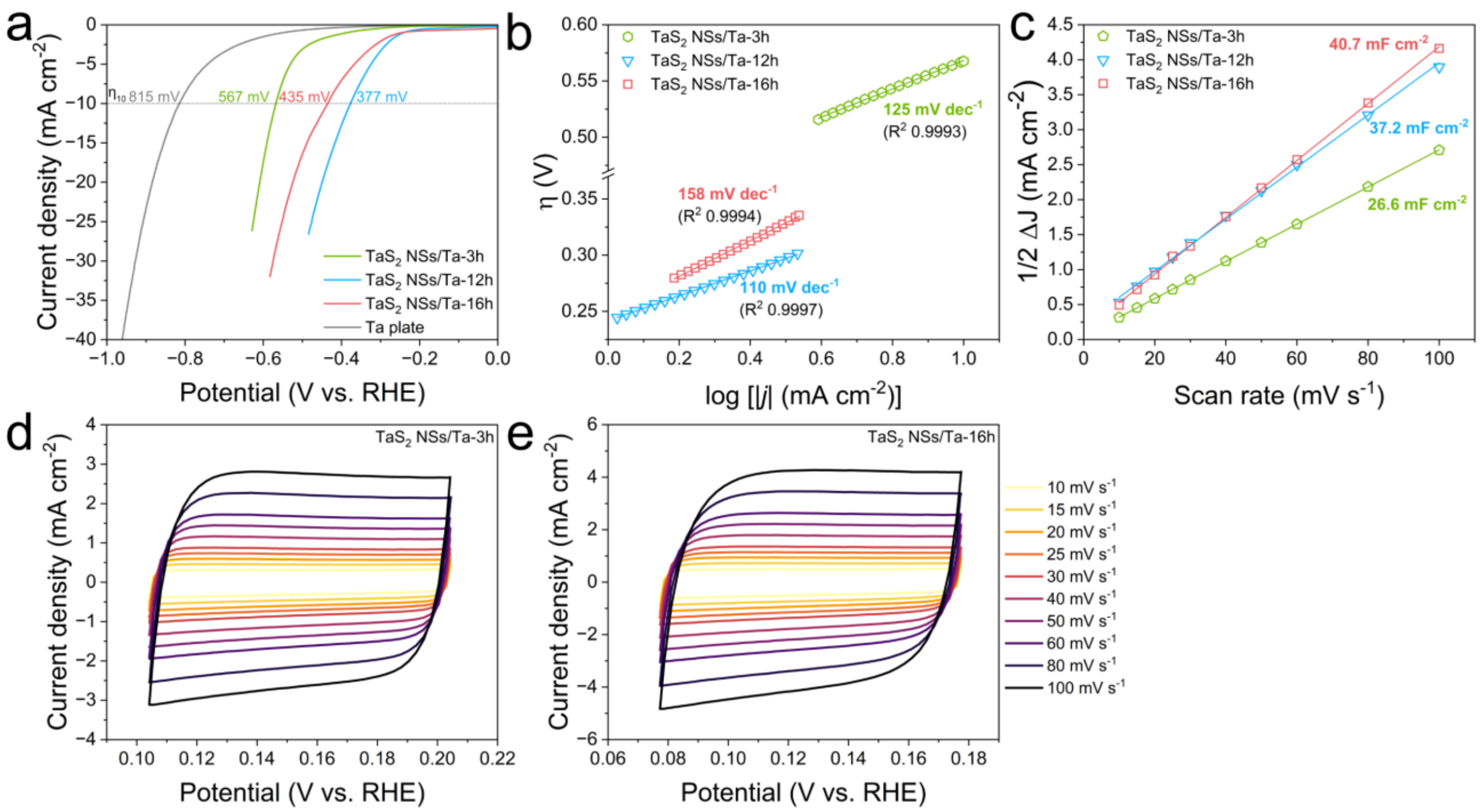

Figure S17. Effect of the reaction time on the HER activity of TaS_2 NSs grown on a Ta plate. (a) Polarization curves of TaS_2 NSs grown on a Ta plate prepared with reaction times of 3-16 h. The η_{10} values are 567, 377, and 435 mV for samples synthesized for 3, 12, and 16 h, respectively, showing that a 12 h reaction time yields the catalyst with the highest activity. The dashed lines indicate the current density corresponding to η_{10}. **(b)** Determination of the Tafel slopes from the LSV curves in (a). **(c)** Determination of the double layer capacitance (C_{dl}) from the linear fit of the variation of current density ($\Delta j/2$) as a function of the scan rates. The corresponding R^2 values are 0.9999, 0.9985, and 0.9995 for the samples prepared for 3, 12, and 16 h. **(d-e)** CV curves obtained with different scan rates from 10 to 100 mV s^{-1} for TaS_2 NSs prepared for (d) 3 h, and (e)16 h. The CV for the sample synthesized for 12 h is shown in Figure S21b.

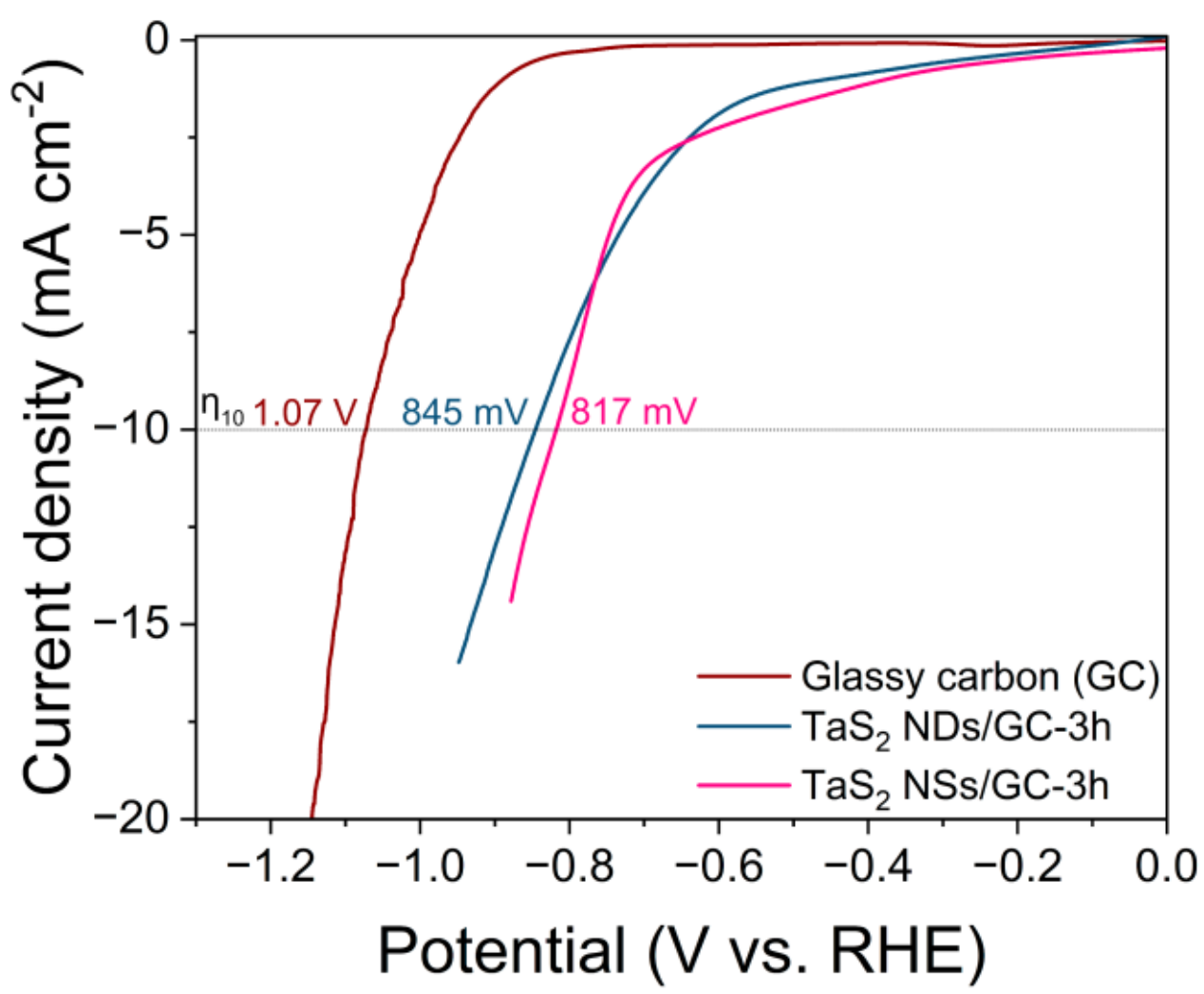

Figure S18. HER activity of TaS_2 nanostructures grown on glassy carbon (GC). LSV curves of TaS_2 NDs/GC (210 °C, 3 h) and TaS_2 NSs/GC (230 °C, 3 h), with a scan rate of 5 mV s^{-1}. The η_{10} values are 845 and 817 mV for the NDs and NSs, respectively, which are better than that of bare GC (1.07 V). However, the TaS_2 film for both morphologies partially detached during HER testing, possibly due to H_2 bubble formation combined with the smooth, polished GC surface, which may limit adhesion between TaS_2 particles and the substrate. Photographs displaying this behavior are shown in Figure S18. This behavior was not observed for TaS_2 nanostructures grown on CC or Ta plate, regardless of the reaction time.

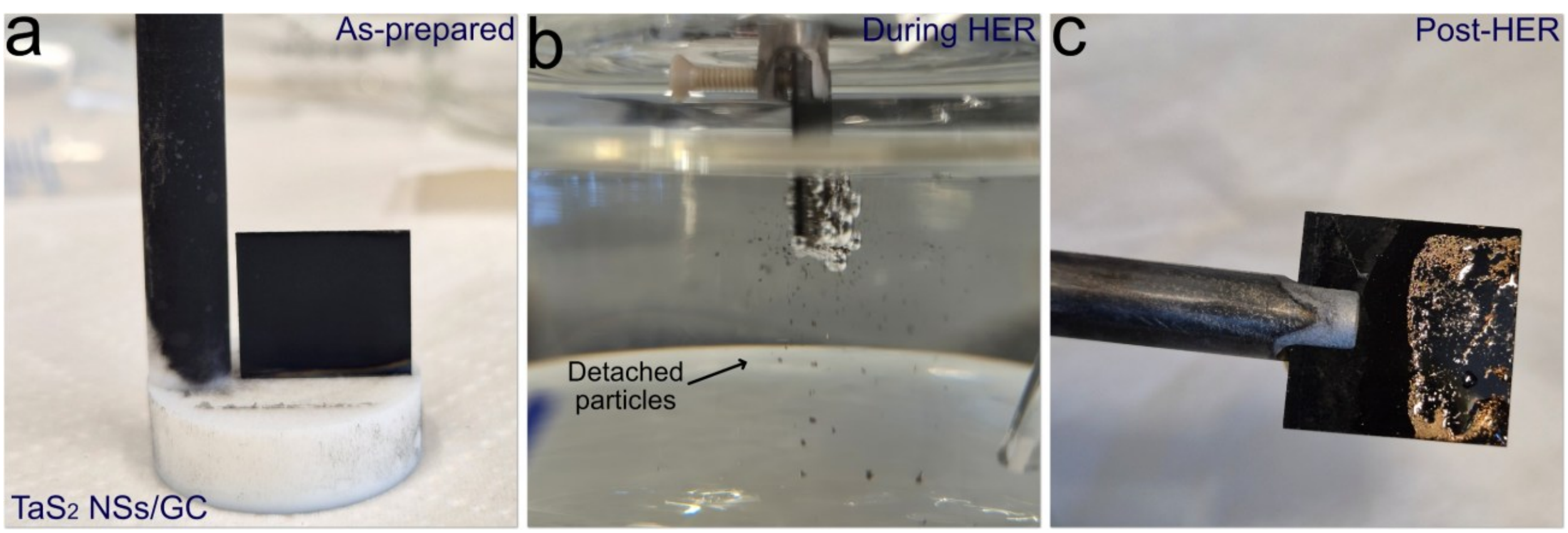

Figure S19. Photographs of the TaS_2 NSs film obtained at 230 °C for 3 h on glassy carbon (GC) at different stages of the HER tests. (a) As-prepared sample after the synthesis and washing procedure, supported in the Teflon holder; **(b)** TaS_2 NSs/GC during the HER tests, showing partial detachment of the particles into the electrolyte during H_2 formation; and **(c)** TaS_2 NSs/GC electrode after the HER tests, exhibiting the preserved TaS_2 film in the region not exposed to the electrolyte, while the region submerged in the electrolyte shows the absence of the film.

Table S2. Overview of the HER performance of TaS_2-based electrocatalysts in acidic medium (0.5 M H_2SO_4)

Label	Electrocatalyst	Synthetic method	η_{10} (mV)	Tafel slope (mV dec^{-1})	Stability	Ref.
**R1**	**Plasma-treated 1T-TaS_2 nanosheets**	Liquid-phase exfoliation	564	135	10 hours @ -0.4 V	3
**R2**	**1T TaS_2 nanosheets**	High temperature solid-state reaction	545	158.9	Not available	4
**R3**	**Bulk 1T-TaS_2**	Solid-state reaction	953*	174*	Not available	5
**R4**	**1T-TaS_2 Nanosheets electrochemically exfoliated**	Solid-state reaction followed by electrochemical and sonication-assisted exfoliation	370	100	10 h @ -0.3 V	6
**R5**	**1T-TaS_2**	Solid-state reaction and electrochemical exfoliation	444	104	Not available	6
**R6**	**1T-TaS_2 vertical nanosheets**	Hot-injection	318	185.4	≈ 30 h @ -0.3 V	7
**R7**	**1T-TaS_2**	Solid-state reaction	770	~95 *	Not available	8
**R8**	**Rectangular TaS_2**	Low-pressure CVD (LPCVD)	302	108	Not available	9
**R9**	**N_2H_4-intercalated 2H-TaS_2**	CVD followed by molecular intercalation	370	76	1 h @ -0.5 V	10
**R10**	**2H-TaS_2 nanosheets**	Arc discharge method	~ 380*	105	Not available	11
**R11**	**6R-TaS_2**	Solid-state reaction	1260	252	Not available	12
**R12**	**3R-TaS_2 nanosheets**	Solid-state reaction	710	40-120	Not available	13
**R13**	**6R-TaS_2**	Solid-state reaction	377	Not available	Not available	14
**This work**	**1T-TaS_2 nanosheets on Ta plate**	Solvothermal	377	110	45 h @ -10 mA cm^{-2}	

*Values estimated from the plots

Supplementary Notes 2: Electrochemical impedance spectroscopy (EIS)

The EIS datasets were fitted using the equivalent circuits shown in **Figure S20b**, comprising either 1 or 2 R-C loops, depending on the sample. Constant phase elements were used instead of ideal capacitors, with their impedance defined as:

$$Z_{CFE} = \frac{1}{Q(j\omega)^{\alpha}}$$

where Q is the CPE parameter, ω is the angular frequency, and α is the exponent describing the deviation from ideal capacitive behavior, with $\alpha = 1$ corresponding to an ideal capacitor. For all fits, α ranged between 0.8 and 1.

The EIS data of the samples grown on the Ta plate (**Figures S20a,c**) and the NSs grown on CC (**Figure S20e**) were fitted with eq. circuit 1, comprising one R-C loop. The EIS data of the TaS_2 NDs on CC (**Figure 20g**) were fitted with eq. circuit 2, comprising two R-C loops to account for an additional high-frequency semicircle. This feature might arise from a charge transfer process at the CC-TaS_2 interface that is not apparent in the other samples. The EIS dataset of the NDs grown on Ta (**Figure S20c**) was fitted with eq. circuit 1, in the frequency range from 50 Hz to 0.1 Hz. The higher-frequency region exhibited features that could not be satisfactorily described by an additional R-C loop. These features may originate from additional processes contributing to the impedance response in the high- and mid-frequency regions, such as ion intercalation or diffusion-related phenomena, the detailed investigation of which was beyond the scope of the present work.

For all samples, the low-frequency region, associated with the HER charge-transfer process, is well described by a single semicircle. Importantly, the inclusion of the additional high-frequency R-C loop does not significantly affect the fitting of the main semicircle or the extracted charge-transfer resistance values. Furthermore, no diffusion-related features were observed in the low-frequency region, indicating that mass-transport limitations do not dominate the HER response under the investigated conditions. Therefore, the discussion focuses exclusively on the low-frequency charge-transfer process relevant to HER activity.

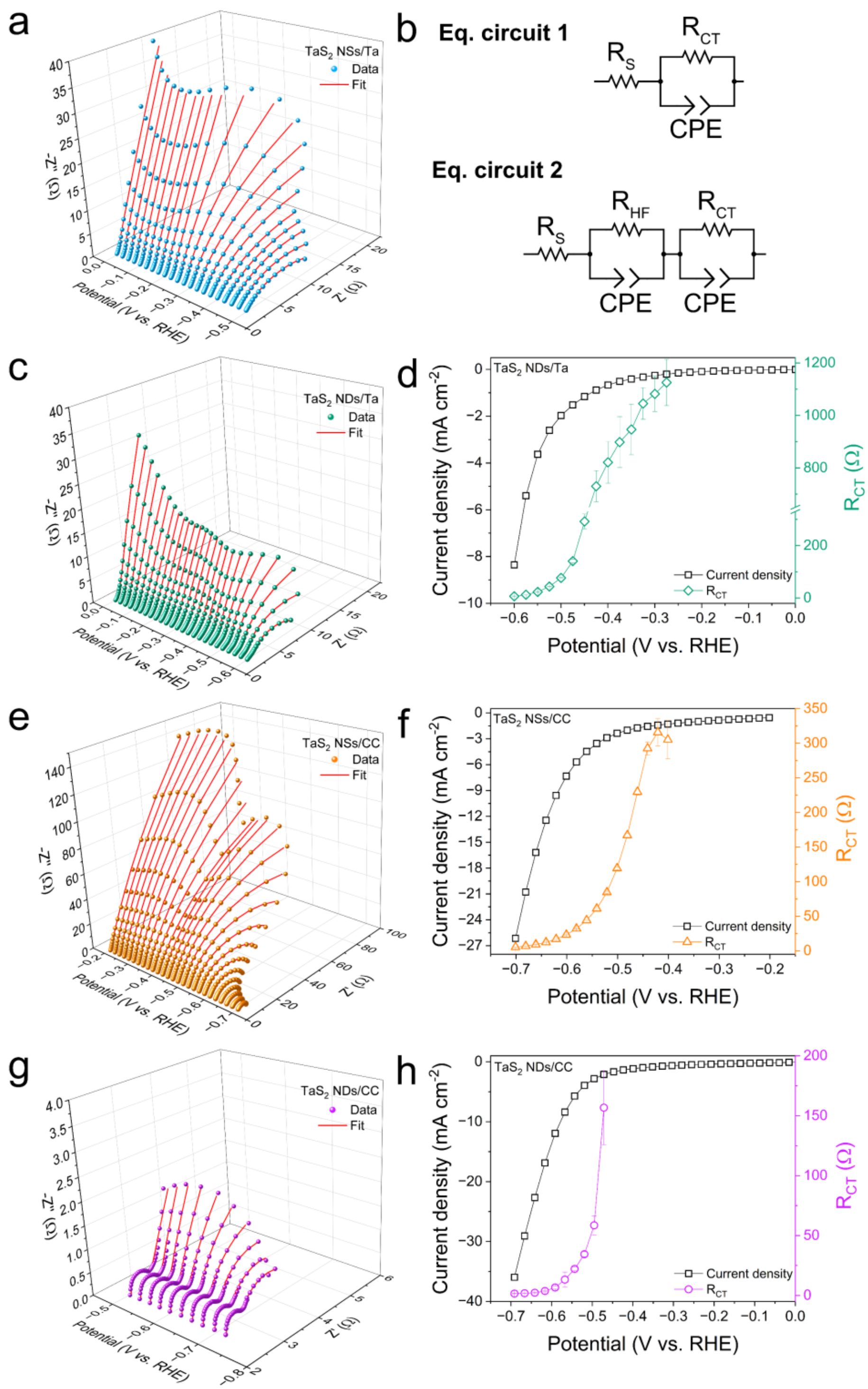

Figure S20. EIS measurements. Nyquist plots for **(a)** TaS_2 NSs/Ta, **(c)** TaS_2 NDs/Ta, **(e)** TaS_2 NSs/Ta, **(g)** TaS_2 NDs/CC, where the EIS data were fitted in the potential range near the onset of HER. **(b)** Equivalent circuit diagrams used for fitting the EIS data. R_S, R_{CT}, and R_{HF} correspond to the series resistance, charge transfer resistance, and high frequency resistance, respectively. CPE stands for constant phase element. Charge transfer resistance (R_{CT}) potential obtained by fitting the EIS data and current density as a function of the applied potential for **(d)** TaS_2 NDs/Ta, **(f)** TaS_2 NSs/Ta, **(h)** TaS_2 NDs/CC.

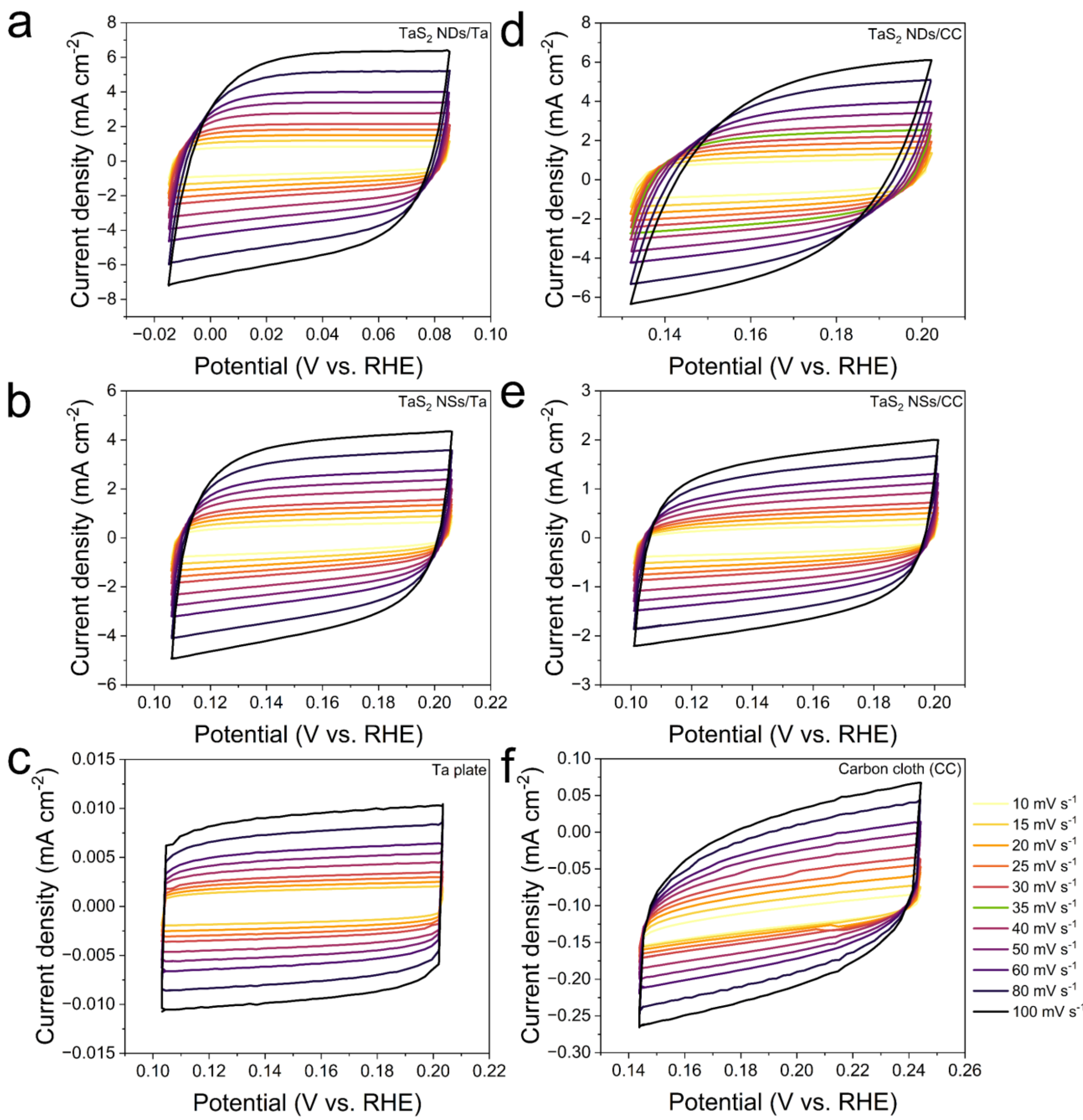

Figure S21. Cyclic voltammetry (CV) curves recorded at varying scan rates (10-100 mV s^{-1}) within a narrow potential window for (a) TaS_2 NDs/Ta, **(b)** TaS_2 NSs/Ta, **(c)** bare Ta plate, **(d)** TaS_2 NDs/CC, **(e)** TaS_2 NSs/CC, and **(f)** bare carbon cloth (CC).

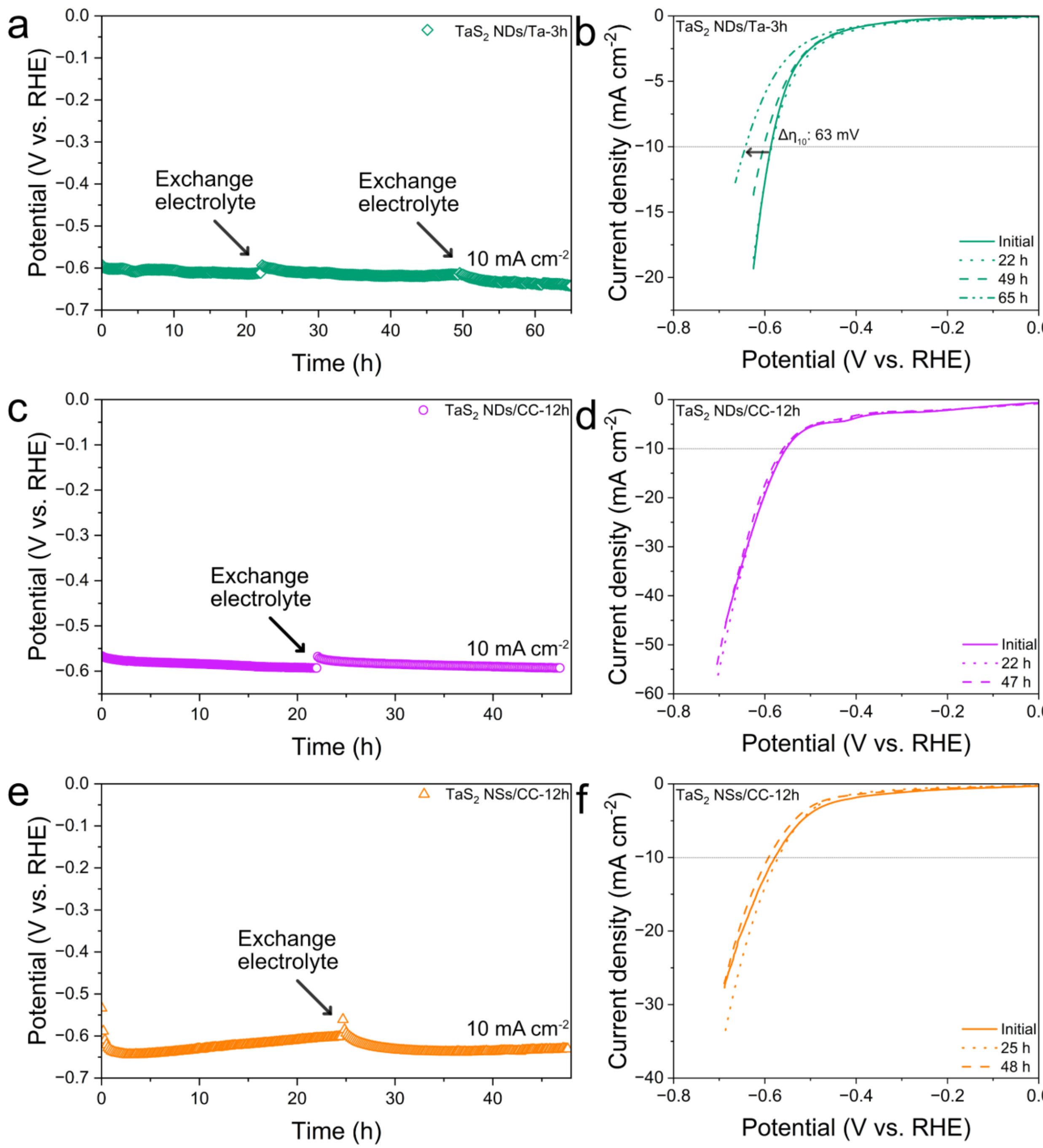

Figure S22. Long-term stability tests of the self-supported TaS_2 electrocatalysts on different substrates. (a-c) Chronopotentiometry measurements performed at a constant current density of 10 mA cm^{-2} for **(a)** TaS_2 NDs/Ta-3h; **(b)** TaS_2 NDs/CC-12h, and **(c)** TaS_2 NSs/CC-12h. **(d-f)** LSV curves recorded before and after the chronopotentiometry tests are shown in (a-c) for **(d)** TaS_2 NDs/Ta-3h; **(e)** TaS_2 NDs/CC-12h, and **(f)** TaS_2 NSs/CC-12h. The TaS_2 NDs grown on a Ta plate, shown in (f), exhibit overlapping LSV curves up to 49 h of testing, followed by an increase in overpotential of 63 mV after 65 h compared to the initial value. In contrast, the TaS_2 NDs and NSs grown on CC show nearly overlapping curves throughout the measurements. The dashed lines in the LSV curves indicate the current density used to determine the overpotential (η_{10}).

Post-electrochemical characterization of TaS_2 nanosheets grown on the Ta plate

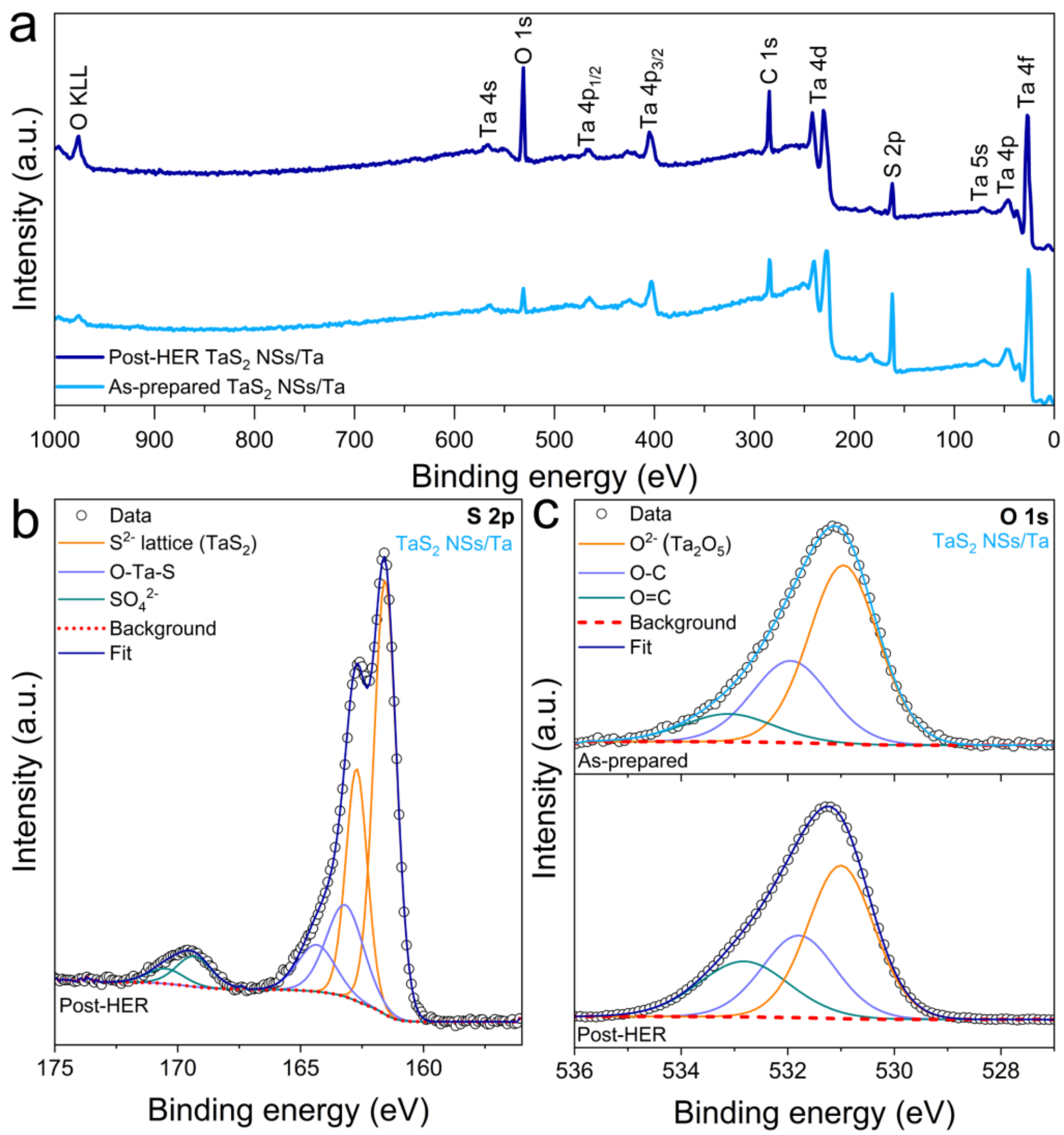

Figure S23. XPS characterization of the TaS_2 NSs/Ta electrocatalysts before and after HER stability tests for 45 h. (a) Survey scan spectra of as-prepared and post-HER, indicating the presence of only Ta, S, O, and C elements. **(b)** S 2*p* core-level XPS spectrum of TaS_2 NSs/Ta after HER tests. Same data as in Figure 4b (main text), presented over an extended energy range. **(c)** O 1*s* core-level XPS spectra of TaS_2 NSs/Ta samples, as-prepared and post-HER. The peak at the lowest binding energy (530.95–531.01 eV) is assigned to the lattice oxygen O^{2-} in Ta_2O_5; the second peak at (531.82–531.95 eV) corresponds to organic C=O, and the peak at 532.85–533.11 eV can be associated with organic C-O. The increase in the intensity of the latter peak after HER tests can be associated with the SO^{4-} presence in the sample.

Table S3. Relative quantification of TaS_2 NSs/Ta samples from XPS survey scans.

Sample	Ta (at%)	S (at%)	O (at%)
**As-prepared**	20.78	66.96	12.25
**After HER**	22.24	29.76	48.01

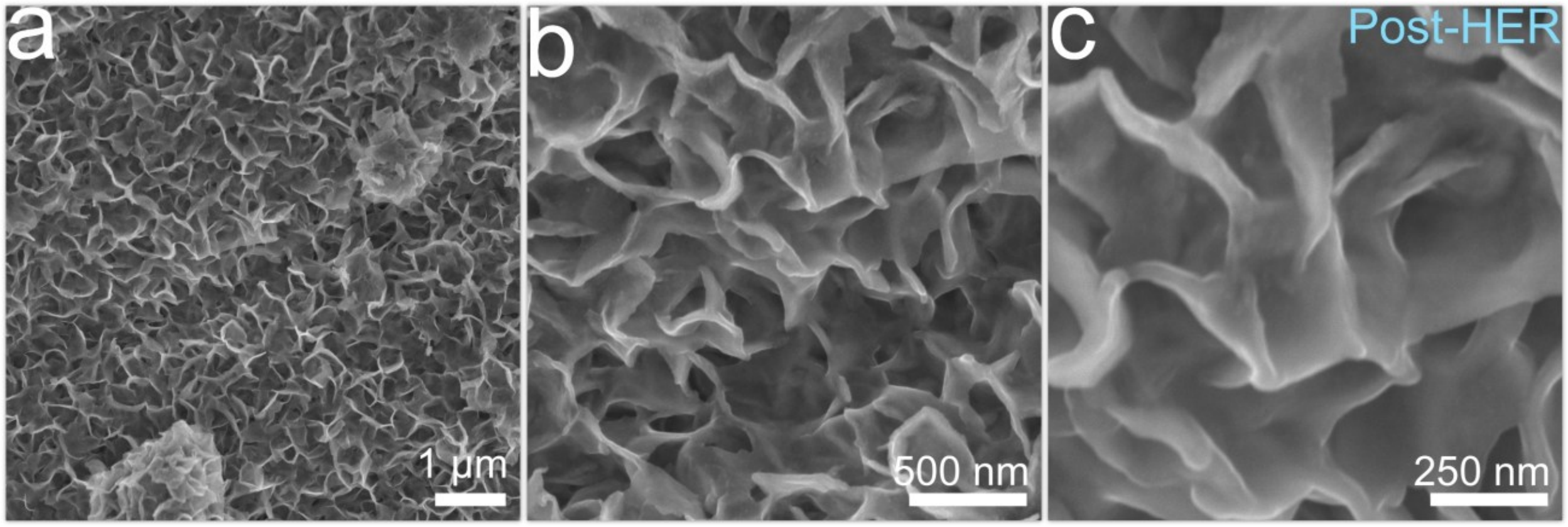

Figure S24. Post-HER morphological characterization of the TaS_2 NSs/Ta electrode after ~45 h of testing. (a-c) SEM images acquired at different magnifications reveal that the crumpled nanosheet morphology is largely preserved after the electrocatalytic tests, with only a slight increase in the thickness of the nanostructures being observed.

Effect of replacing 1-dodecanethiol with tert-dodecanethiol as the sulfur source

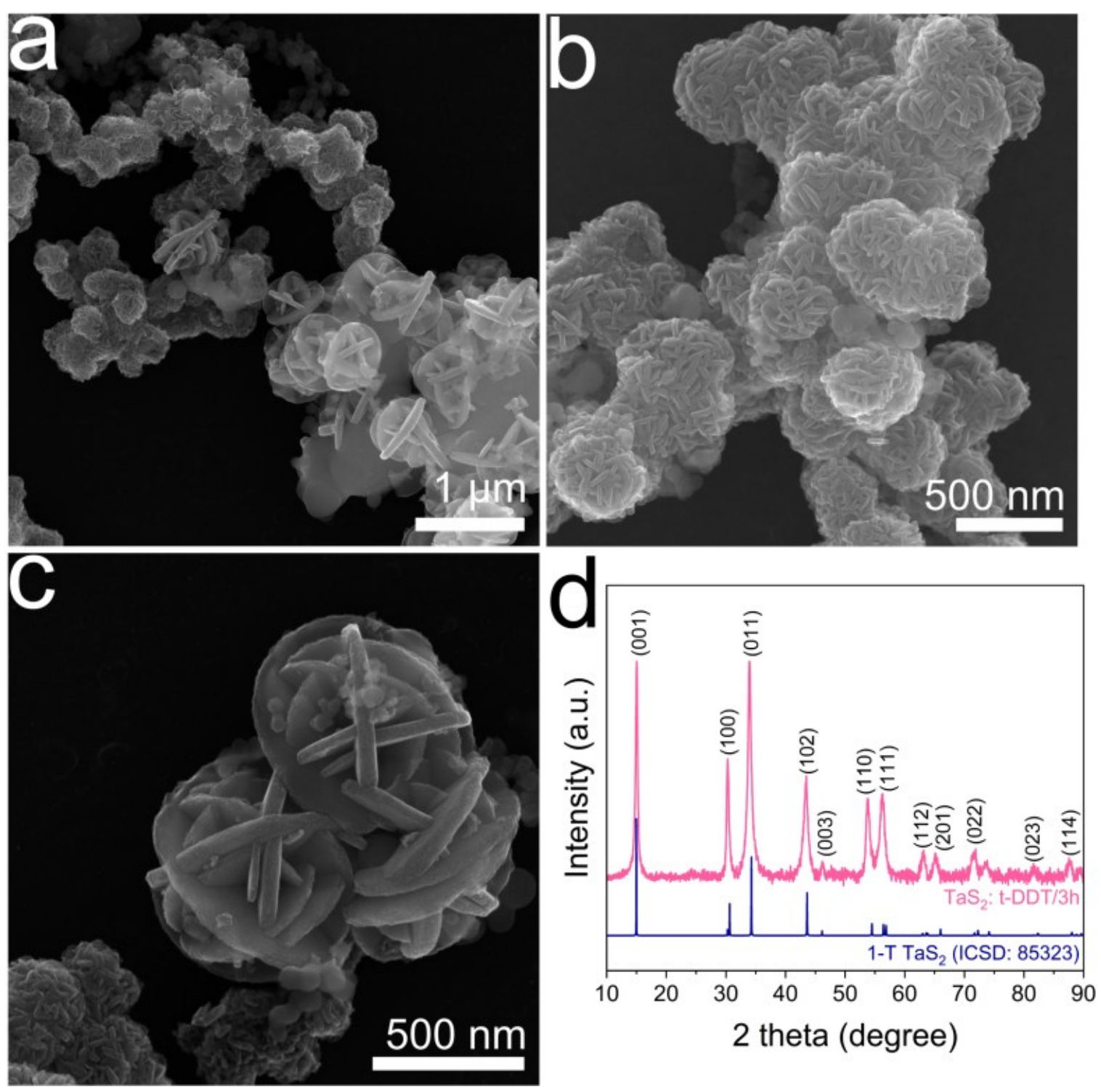

Figure S25. Characterization of the TaS_2 particle powder synthesized using tert-dodecanethiol (t-DDT) as the sulfur source instead of 1-DDT at 210 °C for 3 h. (a-c) SEM images of the TaS_2 particles, showing the formation of assembled nanodisks mixed with smaller plate-like structures, indicating reduced morphological control compared to the synthesis using 1-DDT. **(d)** PXRD pattern of the obtained product, revealing high crystallinity and phase purity, similar to that observed for the material synthesized employing 1-DDT. No substrate was employed in this reaction.

References

1. A. Spijkerman, J. L. de Boer, A. Meetsma, G. A. Wiegers and S. van Smaalen, *Physical Review B*, 1997, **56**, 13757-13767.
2. A. Demont, C. Prestipino, O. Hernandez, E. Elkaïm, S. Paofai, N. Naumov, B. Fontaine, R. Gautier and S. Cordier, *Chemistry – A European Journal*, 2013, **19**, 12711-12719.
3. H. Li, Y. Tan, P. Liu, C. Guo, M. Luo, J. Han, T. Lin, F. Huang and M. Chen, *Advanced Materials*, 2016, **28**, 8945-8949.
4. Y. Feng, S. Gong, E. Du, X. Chen, R. Qi, K. Yu and Z. Zhu, *The Journal of Physical Chemistry C*, 2018, **122**, 2382-2390.
5. X. Chia, A. Ambrosi, P. Lazar, Z. Sofer and M. Pumera, *Journal of Materials Chemistry A*, 2016, **4**, 14241-14253.
6. H. Chen, J. Si, S. Lyu, T. Zhang, Z. Li, C. Lei, L. Lei, C. Yuan, B. Yang, L. Gao and Y. Hou, *ACS Applied Materials & Interfaces*, 2020, **12**, 24675-24682.
7. W. Shen, L. Qiao, J. Ding and Y. Sui, *Journal of Alloys and Compounds*, 2023, **935**, 167877.
8. J. Luxa, V. Mazánek, M. Pumera, P. Lazar, D. Sedmidubský, M. Callisti, T. Polcar and Z. Sofer, *Chemistry – A European Journal*, 2017, **23**, 8082-8091.
9. Y. Feng, K. Yu and Z. Zhu, *CrystEngComm*, 2019, **21**, 3517-3524.
10. Y. Guo, Q. Chen, A. Nie, H. Yang, W. Wang, J. Su, S. Wang, Y. Liu, S. Wang, H. Li, Z. Liu and T. Zhai, *ACS Nano*, 2020, **14**, 1635-1644.
11. I. Raj, Y. Duan, D. Kigen, W. Yang, L. Hou, F. Yang and Y. Li, *Frontiers of Materials Science*, 2018, **12**, 239-246.
12. A. Kagkoura, F. M. Oliveira, K. Mosina, J. Luxa and Z. Sofer, *ACS Applied Materials & Interfaces*, 2025, **17**, 69571-69578.
13. H. G. Shiraz, M. Vagin, Z. U. Khan, R. Chmielowski, R. Crispin and M. Berggren, *International Journal of Hydrogen Energy*, 2025, **100**, 915-920.
14. L. Najafi, S. Bellani, R. Oropesa-Nuñez, R. Brescia, M. Prato, L. Pasquale, C. Demirci, F. Drago, B. Martín-García, J. Luxa, L. Manna, Z. Sofer and F. Bonaccorso, *Small*, 2020, **16**, 2003372.
